# Binge Drinking During Adolescence Induces Long-Lasting Oxidative Stress and Inflammation in the Heart and Promotes Cardiac Dysfunction in Rats

**DOI:** 10.3390/antiox15101360

**Published:** 2026-10-21

**Authors:** Hugo S. Díaz, Katherine V. Pereyra, Karla G. Schwarz, Rodrigo Iturriaga, Rodrigo Del Rio

**Affiliations:** 1 Instituto de Ciencias Biomédicas, Facultad de Ciencias de la Salud, Universidad Autónoma de Chile, Santiago 8580000, Chile; rodrigo.iturriaga@uautonoma.cl; 2 Laboratory of Cardiorespiratory Control, Pontificia Universidad Catolica de Chile, Santiago 8580000, Chile; kvpereyra@uc.cl (K.V.P.); karlaschwarz@uc.cl (K.G.S.); 3 Centro de Excelencia en Biomedicina de Magallanes (CEBIMA), Universidad de Magallanes, Punta Arenas 6200000, Chile; 4 Center for Aging Research and Healthy Longevity, Faculty of Sciences, Universidad Mayor, Santiago 8580745, Chile; 5 Department of Cell Biology and Physiology, School of Medicine, University of Kansas Medical Center, Kansas City, KS 66160, USA

**Keywords:** adolescent binge drinking, oxidative stress, cardiac function

## Abstract

**Background**: It is not well-known whether cardiovascular alterations elicited by adolescent binge drinking (ABD) are reversible or if they persist following long-term abstinence until adult life. Thus, we studied the long-term impact of ABD on oxidative stress, inflammation and cardiovascular function. **Methods**: Twenty-four adolescent Sprague-Dawley rats underwent a controlled or ABD-like protocol, (ethanol 3.00 g/kg i.p.) starting at postnatal day 25 (PND25) to PND38, followed by long-term abstinence until PND70 (adulthood). **Results**: ABD resulted in overt cardiac dysfunction in adult rats. We found a decreased left ventricular compliance as evidenced by a marked reduction in the early-to-late mitral valve blood flow (E/A: 1.23 ± 0.14 vs. 1.51 ± 0.09, control vs. ABD, *p* < 0.05). Importantly, cardiac dysfunction was closely linked to adverse cardiac remodeling and fibrosis in ABD animals compared to control animals. Furthermore, we found that ABD resulted in a significant increase in cardiac arrhythmogenesis. Indeed, ABD rats showed a 2-fold increase in the incidence of cardiac arrhythmias compared to control rats (53.6 ± 12.7 vs. 21.7 ± 5.7 events/h, Control vs. ABD, *p* < 0.05). Finally, we found that cardiac functional impairments were accompanied by increases in cardiac tissue oxidative stress and upregulated levels of pro-inflammatory cytokines (i.e., IL-1β and TNF-α). **Conclusions:** Present results show that ABD drives long-lasting cardiac dysfunction that persists during early adulthood. Oxidative stress and inflammation may contribute to long-term cardiovascular alterations induced by ABD.

## 1. Introduction

Alcohol abuse is the major risk factor of death and disability in the young population being responsible for 5.3% of global deaths [1,2], and the loss of 99.2 million disability-adjusted life years [3]. Alcohol abuse is a major risk factor for cardiovascular and cognitive diseases [2,4,5]. However, the molecular mechanisms by which alcohol exerts its cardiotoxic effects are not clearly understood. Alcohol abuse comprises a wide spectrum of consumption patterns [2], but binge drinking (>60 g of alcohol during a drinking occasion) [6]) is the most harmful for health, being associated with increased risk of all-cause mortality [7]. Alarmingly, adolescents and young adults are more susceptible to binge drinking than adults [8]. A recent systematic review including 173 studies, showed that 38% of adolescents self-reported binge drinking during a period of a month [6]. Most of the studies on adolescent binge drinking (ABD) mainly focused on the risk of developing alcoholism and cognitive dysfunction in early adulthood [7,9], but less attention has been given to cardiovascular alterations following ABD.

It has been shown that acute alcohol intoxication and binge-drinking behavior increases blood arterial pressure in young adults [10,11] and triggers cardiac arrhythmogenesis [10,12]. Also, chronic abuse causes alcoholic cardiomyopathy [10]. Indeed, high doses of ethanol (100 mM) induce oxidative stress, mitochondrial cell membrane depolarization, cytochrome c release, and caspase-3-mediated apoptosis in isolated rat cardiomyocytes [13]. In animal models, ABD produces oxidative stress in cardiac tissue [13,14]. Rodents exposed to ABD had elevated arterial blood pressure and increased levels of cardiac reactive oxygen species (ROS) and nuclear factor-kappa B (NF-κB) linking ABD-triggered cardiac damage with oxidative stress and inflammation [14]. While most of the studies addressed the acute effects of ABD on cardiovascular physiology, much less is known about the chronic long-term effects of ABD on cardiovascular function. Furthermore, whether cardiovascular alterations elicited by ABD during adolescence persist during adult life even when following abstinence is completely unknown.

Accordingly, we studied whether episodic binge-like alcohol exposure during adolescence induces cardiovascular alterations that persist into adult life despite a significant period of abstinence. Also, we aimed to determine whether cardiovascular alterations, if any, are linked to oxidative stress/inflammation in the heart of ABD rats. For this we studied cardiovascular function in adolescent rats (postnatal day 25, PND25), exposed to ABD or control treatment, until PND38 (late adolescence), followed by an abstinence period of 4 weeks (PND70, adulthood).

## 2. Materials and Methods

### 2.1. Adolescent Binge-Drinking-like Protocol

**Figure 1:**
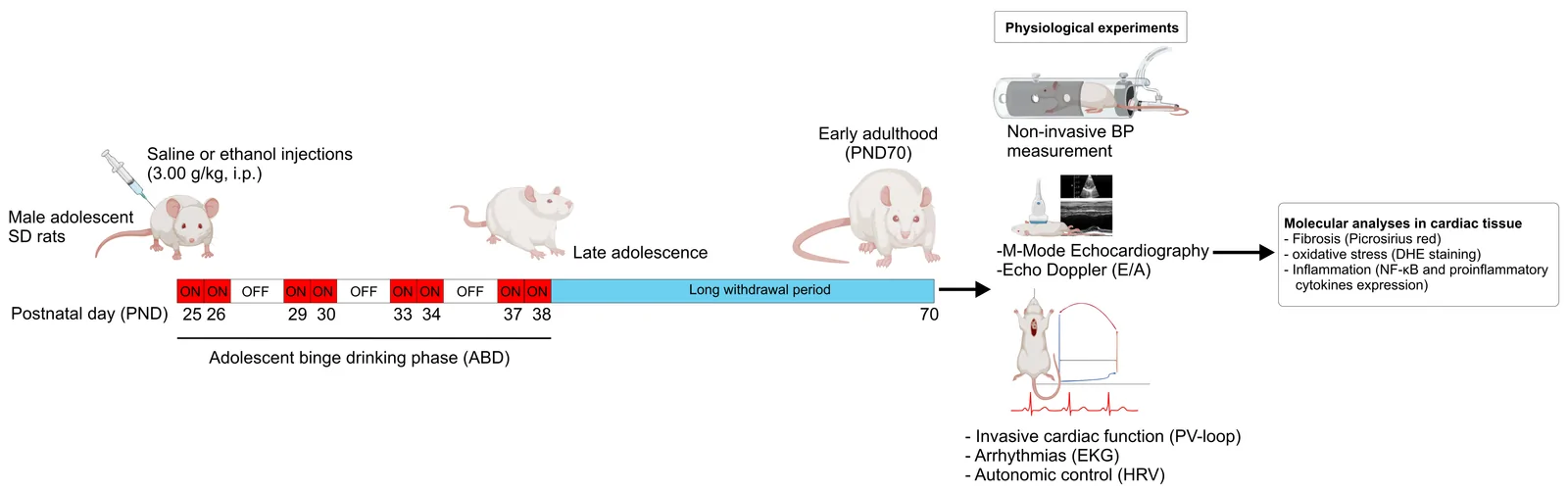
Adolescent binge-drinking protocol. Graphical illustration of the main procedures described in the Methods Section. Sprague-Dawley male rats underwent ABD or control treatment until PND38 (late adolescence), followed by a period of abstinence. A series of physiological and molecular biology molecular measurements were performed according to the illustration and their detailed description is provided in Section 2.

Twenty-four male Sprague Dawley rats at postnatal day 25 (PND 25) were housed in the animal facility of Pontificia Universidad Católica de Chile and maintained on a 12:12 h light-dark cycle at 22 °C, with *ad libitum* access to water and food. The experimental protocol was approved by the Comite Etico Cientifico para el Cuidado de los Animales y el Ambiente from the Pontificia Universidad Catolica de Chile (Protocol ID 201010001) and was conducted according to the National Institutes of Health Guide for the Care and Use of Laboratory Animals. Rats were randomly assigned to control or ABD groups. The ABD rats received ethanol 3.0 g/kg, 25% *v*/*v* in saline solution administrated intraperitoneally (i.p.). As is shown in Fig, 1, the binge-drinking pattern consisted of 2 days of alcohol followed by 2 days without alcohol (2 days-ON, 2 days-OFF) as previously described [15,16,17] and saline treatment consisted of 0.9% NaCl injected i.p. Both treatments were administrated at 25, 27, 29, 30, 33, 34, 37, and 38 PNDs. In this protocol, the maximum blood ethanol concentrations reached 210 ± 11 mg/dL at 30 min post-injection, followed by a gradual decline in the next hours [15,16,17]. After an abstinence period of 32 days (PND70, when rats reached adulthood, end-point physiological measurements were performed. At the end of the experiments, animals were euthanized with an overdose of sodium pentobarbital (100 mg/kg i.p.) (Figure 1).

### 2.2. Non-Invasive Blood Pressure Measurements

Arterial blood pressure (BP) was measured in control and ABD rats using the non-invasive tail-cuff method (Visitech Systems Inc., Apex, NC, USA) [18]. Animals were acclimatized to tail-cuff measurements before acquisition. For each measurement, after 3 initial test measurements (not used for analysis), 10 consecutive measurements were averaged to determine BP. Baseline BP measurements were done at 10:00 am. Systolic BP (SBP), Diastolic BP (DBP), Mean BP (MABP), pulse pressure (PP) and heart rate (HR) were acquired.

### 2.3. Echocardiography

Rats were anesthetized with 2% isoflurane (balanced in O_2_) and underwent transthoracic echocardiography using a Mindray Z6 Vet system at PND25 and PND70. Systolic function was assessed from M-mode images acquired in the parasternal short-axis view. Diastolic function was evaluated in the apical four-chamber view by measuring early (E) and late (A) diastolic transmitral Doppler flow velocities and calculating the E/A ratio. Left ventricular end-diastolic and end-systolic diameters (LVEDD and LVESD, respectively) were measured over three consecutive cardiac cycles. Left ventricular end-diastolic and end-systolic volumes (LVEDV and LVESV) were subsequently calculated using the Tiechholdz formula [19,20]:

(1) $$\mathrm{LVESV}=\frac{7\times {\mathrm{LVESD}}^{3}}{2.4+\mathrm{LVESD}}$$

(2) $$\mathrm{LVEDV}=\frac{7\times {\mathrm{LVEDD}}^{3}}{2.4+\mathrm{LVEDD}}$$

Fractional shortening (FS), ejection fraction (EF%), stroke volume (SV), cardiac output (CO), were calculated accordingly [19,20]:

(3) $$\mathrm{FS}=\frac{\mathrm{LVEDD}-\mathrm{LVESD}}{\mathrm{LVEDD}}\times 100$$

(4) $$\mathrm{EF}\%=\frac{\mathrm{LVEDV}-\mathrm{LVESV}}{\mathrm{LVEDV}}\times 100$$

SV = LVEDV − LVESV(5)

CO = SV × HR(6)

### 2.4. Invasive Cardiac Hemodynamic Assessment

Animals were anesthetized with α-chloralose and urethane (40 and 800 mg/kg, respectively) and intubated with a 16-gauge cannula. Under binocular magnification, a 2-F pressure-volume (PV) conductance catheter (SPR-869,Millar, Pearland, TX, USA) was placed into the right carotid artery and advanced into the LV as previously described [21,22,23]. After an equilibration period (25 min), 10–15 successive PV loops were recorded and used to calculate hemodynamic parameters. Load-independent cardiac function parameters were determined by calculating the slope of the end-systolic pressure-volume relationship (ESPVR) and the end-diastolic pressure-volume relationship (EDPVR). Volumes were calibrated with arterial blood by the cuvette calibration method and a bolus of 30% NaCl (i.v.) was used for determination of parallel conductance [21,22,23]. Data analysis was performed with the PV loop module of LabChart 7.0 software (ADInstruments, Dunedin, New Zealand).

### 2.5. Assessment of Cardiac Autonomic Function

Cardiac autonomic function was evaluated by analysis of heart rate variability (HRV), as previously described [20,23,24]. Heart rate was calculated using *dp/dt* signals derived from continuous blood pressure recordings. Estimations of power spectral density (PSD) of HRV were obtained in a 10 min window, using an autoregressive method analysis after Hann windowing with 50% overlap. Cut-off frequencies were defined as: low frequency (LF_HRV_) 0.04–0.6 Hz and high frequency (HF_HRV_) 0.6–2.4 Hz 8, 28. Frequency-domain heart rate variability (HRV) components were expressed in normalized units (nu) to evaluate the relative distribution of spectral power independently of total variance. To isolate the reciprocal changes between LF_HRV_ and HF_HRV_ bands, the very-low-frequency (VLF, <0.04 Hz) component was mathematically subtracted from the total power TP to obtain the normalized units of LF (LFnu) and HF (HFnu) [25]. We used LF_HRV_/HF_HRV_ ratio as an indicator of cardiac autonomic balance. HRV data analysis was performed using Kubios 3.0.2 software (Finland).

### 2.6. Arrhythmia Incidence

Digital EKG tracings were recorded for all animals during PV-loop experiments using LabChart Pro 2017 software and analyzed for premature ventricular contractions (PVCs), ventricular tachycardia (VT), and ventricular fibrillation (VF), in accordance with the recommendations of the Lambeth Conventions [26,27]. PVCs were identified by the presence of a premature QRS complex; VT was classified as three or more consecutive PVCs and VF was classified as not detectable EKG waveforms and chaotic deflections in blood pressure signal. EKG scoring system G of Curtis and Walker [27] was used for arrhythmia quantifications. Twenty-minute sections of resting EKG were used for arrhythmia incidence. Score G is an equation-based parametric system:

Score G = log (PVCs) + log(VTs)+ 2 × log(VFs) + log(total duration of VF)(7)

All analysis and scoring were performed by two different trained individuals, both blinded to animals’ treatment.

### 2.7. Oxidative Stress Levels

We evaluated the endogenous superoxide levels in the heart by using the fluorescent-dye dihydroethidium (DHE) (Molecular Probes, Life Technologies, Waltham, MA, USA) in frozen cardiac sections. Briefly, after euthanasia, hearts were removed and stored at −80 °C. Then, the heart was sectioned in a cryostat at a thickness of 30 μm and placed on electrostatically charged microscope slides (Superfrost, VWR Scientific, West Chester, PA, USA). Heart sections were incubated with 1 μM DHE in PBS for 30 min at 37 °C. Subsequently, the sections were rinsed three times in PBS for 5 min each and mounted with fluorescent mounting medium (Vector Shield, Vector Laboratories, Newark, CA, USA). Images were acquired using an Olympus CX 31 high-resolution microscope with 10× and 40× objectives (Olympus Corp., Tokyo, Japan). All images were captured under identical conditions of magnification, intensity, brightness, and gain. The images were then analyzed using ImageJ2 software (NIH, Bethesda, MD, USA).

### 2.8. Picrosirius Red Protocol and Hematoxylin/Eosin Staining

At the end of physiological experiments rats were fixed with paraformaldehyde 4% by intracardiac perfusion. The hearts were included in paraffin, and 10 µm transversal sections were obtained using a microtome (RM2125 RTS, Leica, Nussloch, Germany). Heart sections were deparaffinized, rehydrated, and incubated in concentrated picric acid at 60 °C for 30 min, then incubated with picrosirius red (ScyTek Laboratories, SRS250) for 30 min at room temperature. For Heamotoxylin/Eosin staining, heart sections were stained with hematoxylin for 3 min, followed by tap water. After a brief rinse, sections were counterstained with eosin Y for 30 s to visualize cytoplasmic and extracellular structures. Finally, slides were dehydrated through increasing concentrations of ethanol, cleared in xylene, and mounted with Entellan (HX1260033, Merck, Darmstadt, Germany). Optical images of the stained sections were captured at 100 × magnification using a light microscope (Olympus CX31,Tokyo, Japan). Collagen deposition and leukocyte infiltration in myocardial tissue was quantified using Fiji Software (v2.14.0, NIH, Bethesda, MD, USA). For fibrotic index quantification in Picrosirius red images, polarized light images were analyzed. After background subtraction, the positive area for each color was extracted using the “Color Background” function. The following hue ranges were used: 0–9 and 233–255 for red, 10–38 for orange, 39–51 for yellow, and 52–128 for green [28]. The positive area for red color was expressed as a percentage of the total positive area, e.g.,

(8) $${\%}_{\mathrm{red}}=\left({\mathrm{area}}_{\mathrm{red}}/\sum {\mathrm{area}}_{\mathrm{color}}\right)\times 100).$$

In H&E tissue sections inflammation score was evaluated semi-quantitatively across ten randomly selected high-power fields using an ordinal grading scale (Scores 0–4), with Score 0 representing normal architecture with negligible infiltration, Score 1 denotes mild, isolated focal leukocyte clusters (<10% area), Score 2 identifies moderate multi-focal aggregations (10–30% area), and Score 3 marked severe, coalescing infiltrates (30–60% area). Score 4 denotes very severe, diffuse infiltration (>60% area) accompanied by widespread architectural degradation.

### 2.9. Heart Gene and Protein Expression of Inflammatory Biomarkers

Rat hearts were immediately removed after euthanasia, frozen in liquid nitrogen and stored at −80 °C until further processing. RNA isolation was performed using the Trizol^®^ Reagent (15596018 Ambion, Waltham, MA, USA) according to manufacturer’s instructions. RNA purity was assessed by spectrophotometry through the 260/280 ratio (1.78 ± 0.11). Gene expression of target genes was assessed by SYBR green chemistry real-time PCR following reverse transcription of total RNA. Primers’ nucleotide sequences were TNF-α F: AAA TGG GCT CCC TCT CAT CAG TTC; R: TCT GCT TGG TGG TTT GCT ACG AC; IL-1β F: ATG ATG ACG ACC TGC TAG TGT GT; R: TGG CTT ATG TTC TGT CCA TTG AG; IL-6 F: TCC TAC CCC ACC TTC CAA TGC TC R: TTG GAT GGT CTT GGT CCT TAG CG; IL-10 F: GGT TGC CAA GCC TTA TCG GAA ATG R: GCC GCA TCC TGA GGG TCT TC; TGF-β1 F: GTG CGG CAG CTG TAC ATT GAC TTT R: TGT ACT GTG TGT CCA GGC TCC AAA; SOD2: F: CAA GGG AGA TGT TAC AGC R: GGC CTG TGG TTC CTT GCA; NOX2: F: GTG GAC TGG TGT GTG AAT GC R: TTT GGT GGA GGA TGT GAT GA; 18S RNA F: CCT CCA ATG GAT CCT CGT TA; R: AAA CGG CTA CCA CAT CCA AG. Real-time PCR was performed using SYBR Green chemistry (RSMT-B0701, Eurogentec, Belgium) and the AriaMx platform (G8830A, Agilent Technologies, Santa Clara CA, USA). 18S RNA was quantified as an internal control for each sample and quantifications were performed using the 2^−ΔΔCT^ method. Also, the IL-6/IL-10 ratio was made by calculating 2^−(ΔΔCT IL−10−ΔΔCT IL−6)^ for every single sample.

### 2.10. Western Blot

We also analyzed the protein expression levels of cardiac phosphorylated and total p65 NF-κB. Hearts were lysed in RIPA buffer plus protease and phosphatase inhibitor cocktails (1%, P8849 and 4906845001, Sigma, Germany) followed by sonication. Total protein extract concentration was determined using the BCA protein assay kit (ThermoScientific, MA, USA). Proteins were then electrophoresed in polyacrylamide gels (10%), transferred to PVDF membranes (Immobilon-P, Millipore, Burlington, MA, USA) and probed with a mouse polyclonal anti-p-Ser536-p65 (1:3000, bs-0982R, Bioss, Woburn, MA, USA), a mouse monoclonal anti-p65 (1:2000, A-12: sc-514451, Santa Cruz Biotechnology, Dallas, TX, USA) antibody and a rabbit polyclonal anti-β-Actin antibody (1:3.000; #PA5-114817, Invitrogen, Waltham, MA, USA). Primary antibodies were incubated overnight at 4 °C. Membranes were washed in Tris-buffered Saline with 0.1% Tween and incubated for 1 hour (1:3000) with HRP-conjugated goat anti-mouse or rabbit secondary antibodies as applicable (#31460, Invitrogen and #7076, Cell Signaling, Danvers, MA, USA). To accurately quantify the phosphorylation status of p65 (Ser536), identically concentrated protein samples from each subject were resolved and transferred onto two distinct, parallel membranes. Membrane 1 was probed for p-p65, whereas Membrane 2 was probed for total p65. To strictly control for potential inter-gel loading variability, *β*-Actin was assessed on both membranes as an internal loading control. Following the chemiluminescent detection of the primary targets (p-p65 or total p65), horseradish peroxidase (HRP) conjugate inactivation was achieved by incubating the membranes in 300 mM H_2_O_2_ for 30 min to avoid protein loss following harsh stripping protocols. Subsequently, the membranes were reprobed for *β*-Actin. Finally, the relative activation of the NF-κB pathway was determined by calculating the ratio of the normalized phosphorylated fraction to the normalized total protein fraction, expressed as:

$$p65\;\mathrm{phosphorylation}=\frac{p-p65(\mathrm{Ser}536)/{\mathrm{\beta Actin}}_{\mathrm{Membrane}1}\;}{p65(\mathrm{total})/{\mathrm{\beta Actin}}_{\mathrm{Membrane}2}}$$

### 2.11. Multivariable Analysis

Data from all subjects was organized into a 24 × 29 matrix, with 24 rows indicating observations (subjects) and 29 columns representing physiological and molecular variables (dimensions). Only continuous variables were included for multiple correlations and PCA. Correlation matrix was generated from computed Pearson r values for every pair of datasets using GraphPad Prism 9 software (Supplementary Table S2). Hierarchical clustering and heat map creation were performed in Orange 3.40.0 software, which was also used for visualization of multiple variables in 2D plots. Unsupervised PCA was performed by eigenvalue decomposition to obtain its eigenvectors, that were sorted by variances (eigenvalues) in decreasing order. Only the Principal Components (PC) that shown eigenvalues higher than 1 were considered as significant, according to the Kaiser’s rule [22]. After that, the original dataset was transformed using the eigenvectors as weighting coefficients to obtain their respective PC scores as previously described [22]. After PCA, biplots were created to visualize the relationship between the variables in the space of selected components. PCA and biplot scheming were performed using the PCA toolkit of GraphPad Prism 9 software (La Jolla, CA, USA). Also, PC Regression (PCR) was performed using as regressor DHE staining, for assessing the predictive value of oxidative stress for physiological and molecular variables (Supplementary Table S3).

### 2.12. Statistical Analysis

GraphPad Prism 9.0 statistical software (La Jolla, CA, USA) was used to analyze the data. Normal distribution of the data was assessed with the D’Agostino-Pearson test. The statistical significance of the data with normal distribution was evaluated using the *t*-tests (2 groups at the same experimental time) or two-way ANOVAs with repeated measures (2 groups and different time points). Statistical significance of non-normally distributed data was evaluated using nonparametric tests such as Mann–Whitney U (2 groups at the same experiment time) test and two-way ANOVAs followed by a Dunn post hoc analysis. The level of significance was defined as *p* < 0.05. Results are shown as mean  ±  SEM in text and tables and mean  ±  range in figures. Prior to inferential analysis, data were evaluated to ensure they met the underlying assumptions of the proposed statistical models. The assumption of normality was assessed using the Shapiro–Wilk test. For comparisons between two independent groups, the assumption of homoscedasticity (equality of variances) was formally tested using the F-test. When the assumption of equal variances was met, comparisons were performed using the standard unpaired Student’s *t*-test. However, in cases where this assumption was violated (F-test *p* < 0.05), an unpooled variance approach was adopted, and Welch’s *t*-test was utilized to prevent Type I error inflation.

## 3. Results

### 3.1. Arterial Blood Pressure and Echocardiographic Findings Following Binge-Drinking and Abstinence in Adulthood

**Figure 2:**
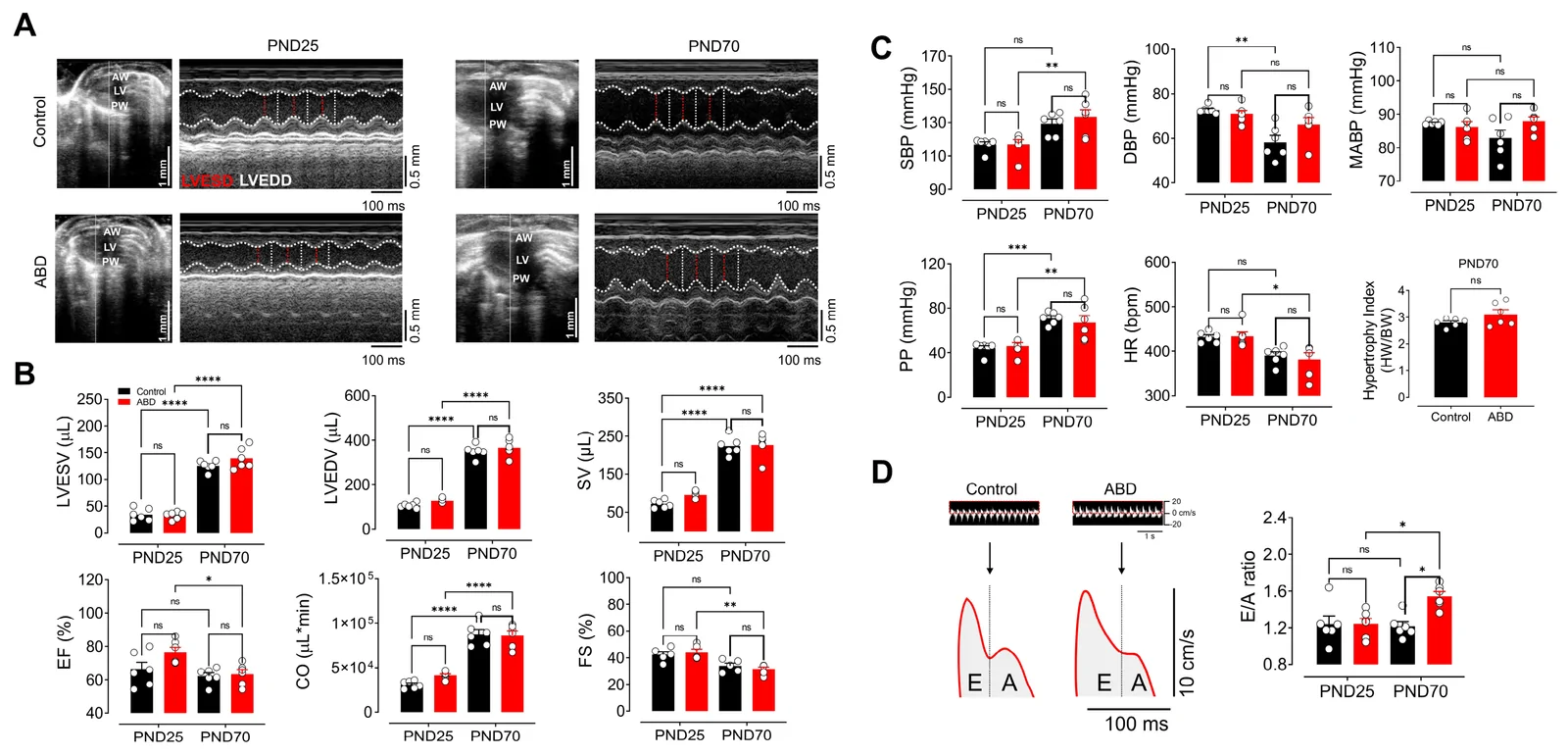
Effect of ABD on arterial blood pressure and non-invasive cardiac function. (**A**) Representative echocardiography images obtained in one control and one ABD rat at PND25 and PND70. (**B**) Longitudinal echocardiographic analyses of cardiac architecture, ejection fraction and heart rate in control and ABD rats. (**C**) Non-invasive pressure measurements at PND25 and PND70, and effect of ABD on cardiac hypertrophy index. (**D**) Diastolic function measurements by Doppler echocardiography before and after ABD exposure. Two-way ANOVA with repeated measurements. Pairwise comparisons indicate (non)significant statistical differences between the ABD and control groups at the same time point, as well as when comparing each group to its own baseline at PND25. Significance brackets span between the origin and destination groups for each specific pairwise contrast. *: *p* < 0.05 and **: *p* < 0.01, ***: *p* < 0.001, ****: *p* < 0.0001, ns: not significant (n = 6 animals per group).

Figure 1 summarizes all the experimental procedures performed in this study. ABD did not cause significant changes in weight gain (173.50 ± 17.82 vs. 179.30 ± 14.46, ABD vs. control), survival (100% both groups) and pulmonary congestion (4.78 ± 0.39 vs. 4.51 ± 0.20, ABD vs. control) compared to control animals. We found no changes in cardiac chamber dimensions nor in stroke volume or cardiac output between ABD and Control animals (Figure 2A,B). Accordingly, non-invasive arterial blood pressure recordings showed no changes in SBP, DBP, MABP, PP and cardiac hypertrophy index in ABD rats when compared to control rats at PND70 (Figure 2C). Contrarily, we found that ABD animals displayed significant functional cardiac alterations (Figure 2D). Indeed, diastolic function was markedly impaired in the ABD group. Specifically, ABD rats exhibited a significantly increased E/A ratio compared to controls (1.23 ± 0.14 vs. 1.51 ± 0.09, control vs. ABD, *p* < 0.05) without significant changes in the left ventricle ejection fraction or fractional shortening (Figure 2D).

### 3.2. Hemodynamic Diastolic Dysfunction

**Figure 3:**
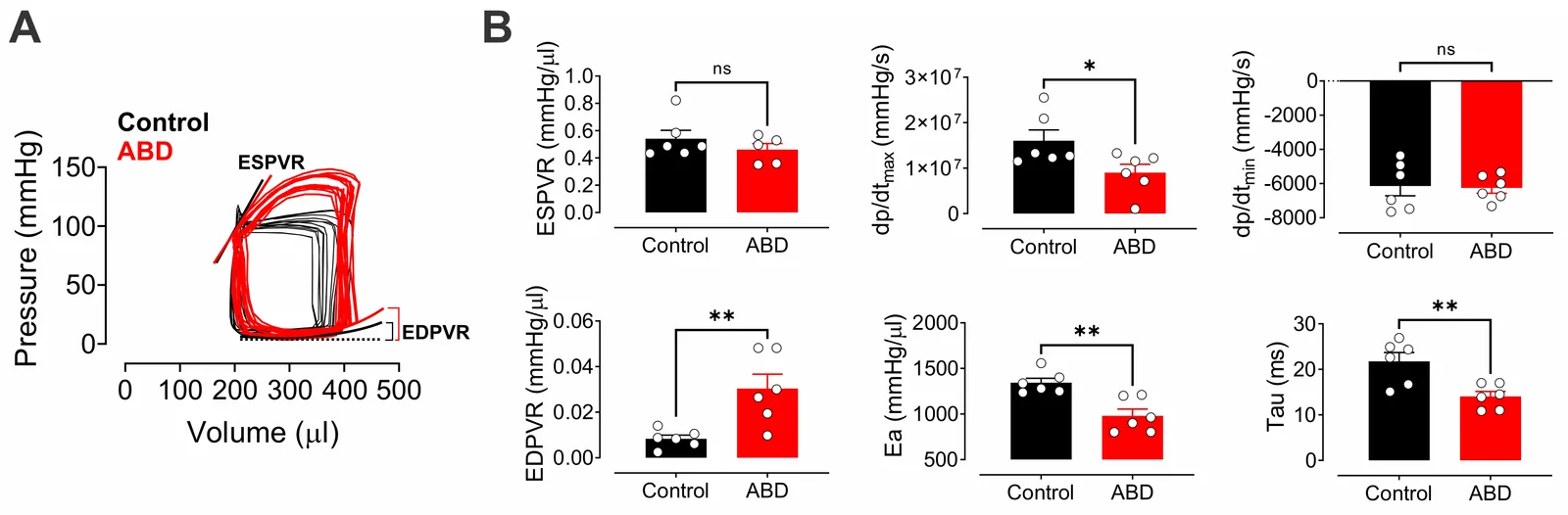
Effect of ABD on cardiac function. (**A**) Representative pressure-volume (PV) loops of one ABD and one control rat at PND70. End systolic pressure-volume relationship (ESPVR and End diastolic pressure-volume relationships curves are depected (**B**) Summary data of cardiac elastance (Ea). Systolic cardiac function (ESPVR, dp/dt_max_) and diastolic function parameters (EDPVR, dp/dt_min_) in 2 groups Unpaired *t*-test, *: *p* < 0.05, **: *p* < 0.01, ns: not significant (n = 6 animals per group).

To obtain load-independent indices of cardiac contractility and compliance, and to mechanically validate the echocardiographic findings, we performed invasive PV loop analyses via left ventricular catheterization in anesthetized rats (Figure 3). The hemodynamic profiles confirmed that systolic parameters such as the end-systolic pressure-volume relationship (ESPVR), and maximal derivative of left ventricular pressure (dp/dtmax) were unchanged in ABD rats compared to control rats (Figure 3B). On the contrary, diastolic function was severely compromised in ABD rats. Indeed, ABD rats showed overt signs of reduced left ventricular compliance as evidenced by a significant increase in the end-diastolic pressure-volume relationship (EDPVR) compared to control rats (0.009 ± 0.001 mmHg/µL vs. 0.0303 ± 0.006 mmHg/µL, control vs. ABD, *p* < 0.05) (Figure 3B).

### 3.3. Chronic Autonomic Imbalance and Arrhythmogenesis

**Figure 4:**
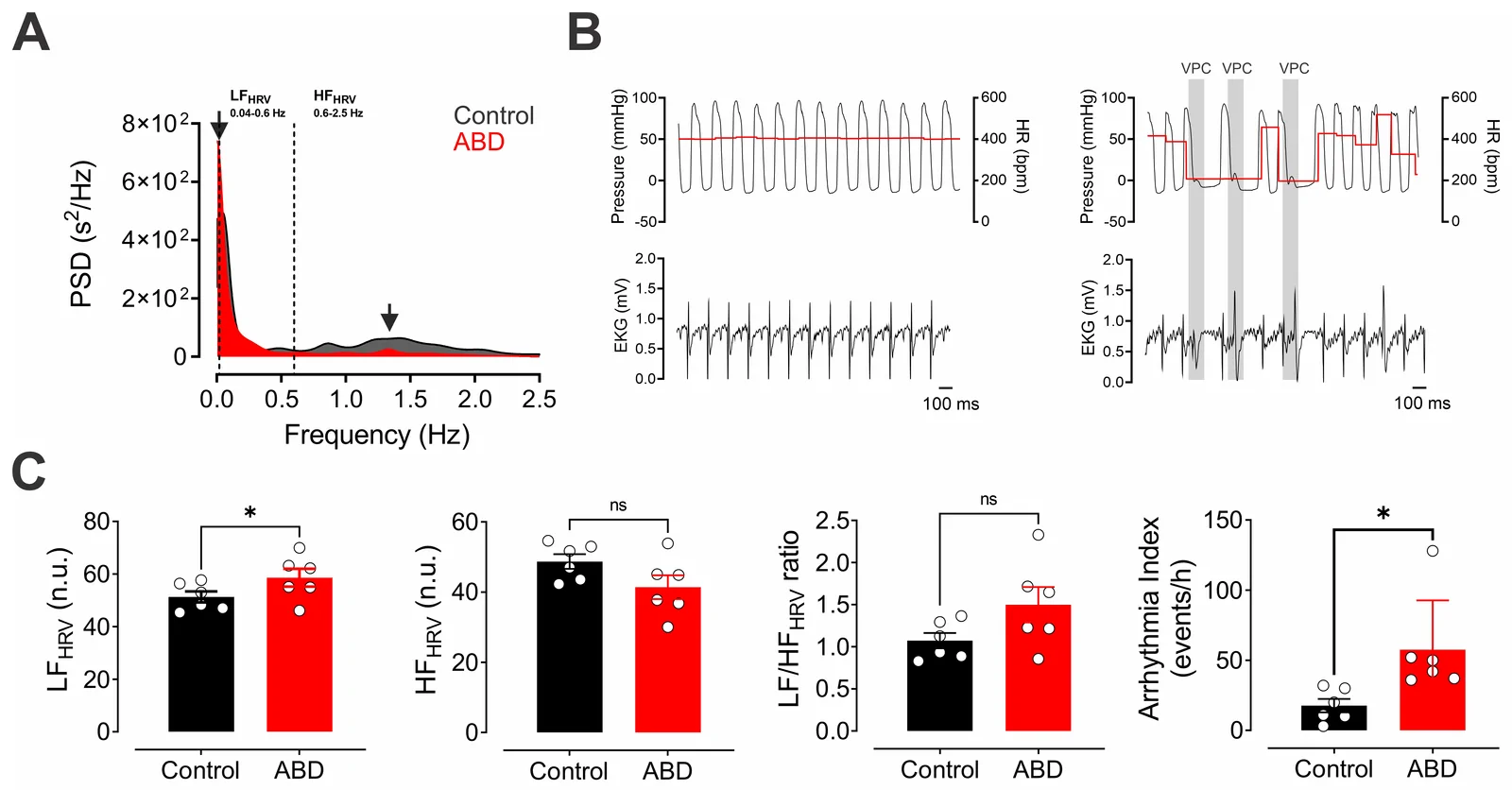
Effect of ABD on cardiac arrhythmogenesis and autonomic control. (**A**) Representative heart rate variability (HRV) autoregressive spectrograms obtained in one control and one ABD animal at PND70. Arrows point to LF_HRV_ and HF_HRV_ peaks. (**B**) Representative traces of arterial blood pressure and EKG recording of one control and one ABD rat at PND70. (**C**) Quantification of HRV parameters, including low and high frequency components (LF and HF respectively), as well as autonomic balance, expressed as the LF/HF ratio. Arrhythmias were classified and scored using a logarithmic scale. Unpaired *t*-tests, *: *p* < 0.05 vs. control, ns: not significant. (n = 6 animals per group).

We investigated the impact of ABD on cardiac autonomic nervous system regulation and cardiac electrical rhythm stability. Sympatho-vagal balance was estimated using power spectral analysis of the heart rate variability (HRV) (Figure 4). HRV spectral analysis showed a shift toward the sympathetic dominant low frequency component (LF) over the high parasympathetic frequency component (HF) in the ABD group compared to the control group (Figure 4C). The presence of cardiac autonomic imbalance was quantitatively reflected by a significant increase in the low- to high-frequency ratio (LF/HF_HRV_ ratio: 1.01 ± 0.08 vs. 1.59 ± 0.23, control vs. ABD, *p* < 0.05) (Figure 4C).

EKG monitoring demonstrated that ABD rats exhibited a significantly (*p* < 0.05) higher frequency of spontaneous cardiac arrhythmias (53.6 ± 12.7 events/h) compared to control rats (21.7 ± 5.7 events/h) (Figure 4C). ABD did not significantly alter the RR, PR, QT and QTc intervals, but induced a marked widening of the QRS complex compared to control (0.03 ± 0.00 s vs. 0.06 ± 0.02 s, control vs. ABD, *p* < 0.05). Furthermore, we found that ABD animals showed a 2-fold increase in the incidence cardiac arrhythmias compared to control animals (Figure 3B). Indeed, compared to control rats, ABD rats showed a 2-fold increase in the arrhythmia G-Score (2.39 ± 0.59 vs. 4.80 ± 0.81, control vs. ABD, *p* < 0.05, Supplementary Table S1).

### 3.4. Cardiac Molecular Biology: Fibrosis, Oxidative Stress, and Inflammation

**Figure 5:**
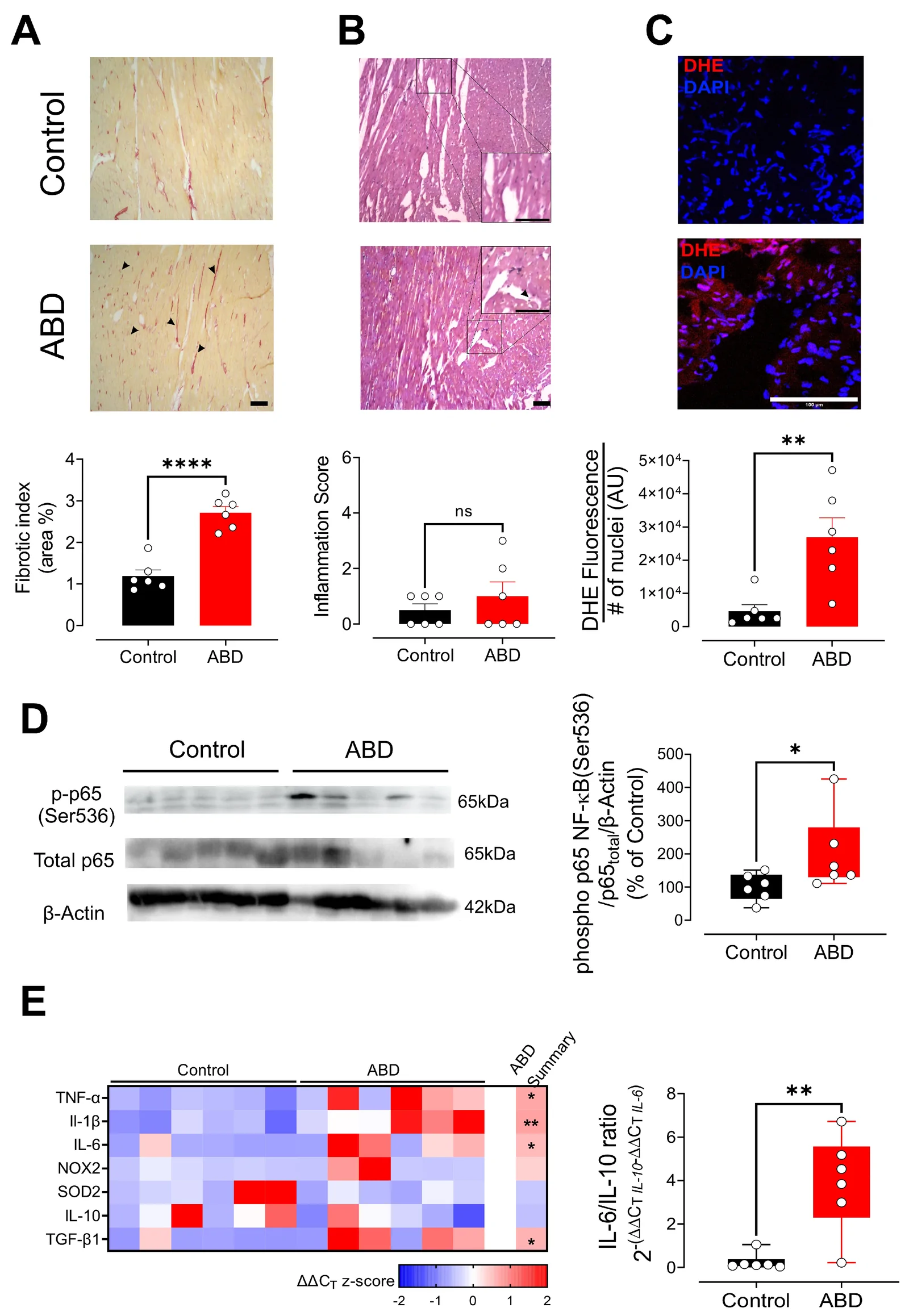
Effect of ABD on cardiac fibrosis, oxidative stress and inflammation. (**A**) Representative Picrosirius red staining micrographs and quantifications of adult control and ABD rats. Triangles show zones of myocardial collagen accumulation. (**B**) Hematoxilin/Eosine staining micrographs and quantifications. Triangles represent inflammatory score 1: small, sparse, focal infiltrates of inflammatory cells (**C**) Representative DHE Staining micrographs and quantifications of DHE fluorescence. (**D**) Representative blots and quantification of protein levels of phosphorylated and total p65 NF-kB. (**E**) Z-score standardized cardiac mRNA expression profile of inflammatory, oxidative, and fibrotic markers in all control and ABD rats, alongside the cardiac inflammatory index derived as 2^−(ΔΔCT IL−10-ΔΔCT IL−6)^. *: *p* < 0.05, **: *p* < 0.01, ****: *p* < 0.0001, ns: not significant (n = 6). Scale bars: 100 µm.

Cardiac extracellular matrix remodeling was assessed using Picrosirius red staining, which allows for the specific quantification of collagen deposition. Optical imaging and subsequent quantification demonstrated a significant accumulation of collagen fibers within the interstitial spaces of the left ventricle in the heart of ABD rats compared to control rats (Fibrotic Index: 2.71 ± 0.37 vs. 1.19 ± 0.15, ABD, vs. control, *p* < 0.0001, Figure 5A). ABD did not promote leukocyte infiltration into the myocardium (inflammation score: 0.50 ± 0.22 vs. 1.00 ± 0.51, ABD, vs. control, Figure 5B). The fibrotic remodeling was tightly coupled with a pro-oxidant and pro-inflammatory myocardial microenvironment. Indeed, dihydroethidium (DHE) fluorescence revealed upregulated levels of reactive oxygen species in the cardiac tissue of adult ABD rats (DHE staining: 4630 ± 1961 vs. 26,859 ± 5878 a.u., control vs. ABD, *p* < 0.01, Figure 5C). Concurrently, the expression of phosphorylated p65 (Ser536) relative to total p65 and β-Actin was significantly increased in the adult ABD cardiac tissue (200 ± 48.0% vs. 100 ± 16.84%; ABD vs. control, *p* <0.05, Figure 5D), confirming the persistent transcriptional activation of inflammatory genes. RT-qPCR analysis of myocardial homogenates demonstrated a significant overexpression of mRNA levels of tumor necrosis factor-alpha (TNF-α), interleukin-1 beta (IL-1β) and interleukin-6 (IL-6) (IL-1β: 2.92 – fold increase in ABD; TNF-α: 1.77 – fold; IL-6: 2.53 – fold increase in ABD, *p* < 0.05, Figure 5E, Detailed RT-qPCR data in Supplementary Figure S1 and Supplementary Table S4). Conversely, the mRNA levels of anti-inflammatory cytokine Interleukin-10 (IL-10) showed a non-significant downward trend (0.74 – fold decrease, *p* = 0.052), resulting in a profoundly elevated IL-6/IL-10 ratio (3.91 ± 0.90 vs. 0.25 ± 0.16; ABD vs. control; *p* < 0.01), indicative of a systemic inflammatory shift. Consonant with the observed reactive interstitial fibrosis, Transforming Growth Factor-beta 1 mRNA (TGF-β1) was significantly upregulated in ABD rats (1.14-fold increase in ABD, *p* < 0.05). Detailed information of RT-qPCR expression can be found in Supplementary Figure S1.

### 3.5. Multivariable Correlations and Principal Component Analysis (PCA)

**Figure 6:**
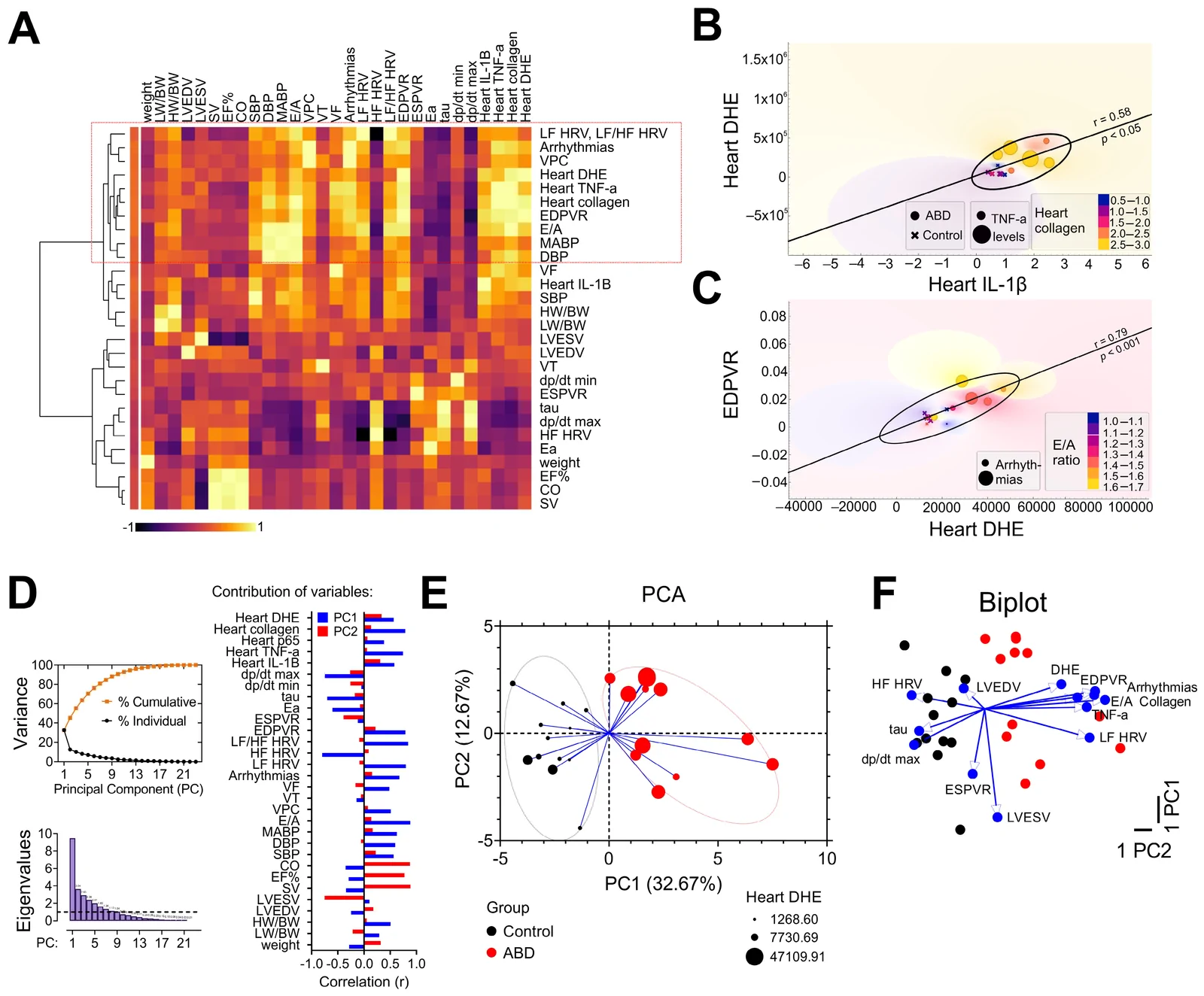
Multivariable analysis, hierarchical clustering and principal component analysis (PCA). (**A**) Unsupervised hierarchical clustering and heat maps of multiple variable correlations. Heat map depicts Pearson-r values. (**B**) Correlation between heart IL-1β and heart DHE levels showing collagen levels in color scale and IL-1β levels in size scale. X represents control animals and dots represent ABD animals and symbol size represent values of TNF-α mRNA levels (**C**) Correlation between heart DHE levels and EDPVR showing collagen E/A ratio in color scale and arrhythmia incidence in size scale. X represent control animals and dots represent ABD animals and symbol size represent arrhythmia incidence (**D**) PCA quality control parameters (individual/cumulative variance and eigenvalues), (**C**,**D**) show Pearson r values and confidence ellipses. (**E**) PC1 and PC2 scores obtained from every data sample. (**F**) Biplot showing distinct cohort clustering: Control animals (black) align with physiological compliance (dP/dt_max_, tau, HF HRV), whereas ABD animals (red) shift along PC1 toward pathological remodeling, oxidative stress, and inflammation (Collagen, DHE, TNF-α, EDPVR, Arrhythmias).

To explore the association between oxidative stress and inflammation with parameters of ABD-driven cardiovascular dysfunction, we computed Pearson r correlation coefficients for each pair of values and later performed unsupervised hierarchical clustering and then plotted them in multivariable correlation matrix (Figure 6A, Supplementary Figure S2, Supplementary Table S2). Based on visual exploration of heatmap, one branch of strongly related variables was found (Pearson-r > 0.5, *p* < 0.05) with leaves including heart DHE, heart collagen (fibrotic index), LF_HRV_, LF/HF_HRV_, Arrhythmias, EDPVR, E/A, MABP and DBP (Figure 6A). In fact, DHE levels were strongly related to fibrotic index (Pearson-r = 0.72, *p* < 0.0001, note color area clusters of heart collagen, Figure 6B) and pro-inflammatory cytokine expression (Figure 6C). To confirm the predictive value of oxidative stress for describing ABD pathophysiology, a principal component regression (PCR) was performed using heart DHE as the regressor, showing *p* < 0.05 for predicting E/A, LF_HRV_, EDPVR, Ea, tau, TNF-α and heart collagen (Supplementary Table S3). Accordingly, PCA reflected two well-differentiated clusters of points after dimensional reduction in data (Figure 6D,E). Loadings biplot showed the contribution of relevant pathophysiological variables to PC1 and PC2, confirming the predictive value of PCA and PCR to describe ABD pathophysiology (Figure 6F).

## 4. Discussion

Present results provide evidence supporting that adolescent ABD-induced cardiac alterations that persisted until early adulthood in rats. The novelty of our experimental design resides in the evaluation of ABD alterations following a long abstinence period, spanning from the end of the adolescence (PND38) to early adulthood (PND70). Unlike most toxicological protocols that assess the acute effects of ethanol intoxication or chronic uninterrupted exposure in adult subjects, our longitudinal approach unmasks a metabolic memory effect. The vast majority of experimental paradigms are heavily restricted to acute exposure phases or immediate post-binge withdrawal windows, typically assessing rodents between PND 37 and 45. While prior investigations have successfully identified transient shifts in tissue damage markers, early hypertrophic remodeling, and acute neuroinflammatory spikes—such as central and peripheral cytokine fluctuations reported by early-stage models [15,29], immediate post-exposure redox imbalances [14,30,31,32], and early adolescent structural alterations [33]—they fundamentally fail to track the ultimate physiological fate of these subjects into early adulthood. Furthermore, alternative paradigms utilizing short-term adult binge protocols [29,34] or molecular tracking confined strictly to adolescence [29] highlight age-dependent discrepancies that emphasize how short-term stimuli fail to capture permanent structural remodeling. By extending our experimental timeline to incorporate a prolonged 32-day abstinence period and evaluating subjects at PND 70, our model shatters these temporal limitations, demonstrating that unlike acute or short-term models where parameters normalize, adolescent exposure triggers a persistent, uncompensated phenotype that defines a distinct late-stage pathophysiological trajectory. Our data confirms that ABD generates permanent molecular and physiological alterations characterized by myocardial oxidative stress, inflammation, and adverse remodeling resulting in diastolic rigidity.

From a sociological and health policy perspective, experts warn of the deeply concerning prevalence of early-onset alcohol consumption (14–16 years) and the severe lack of knowledge regarding cardiovascular risk [35]. Importantly, it has been shown that substance use in the youth acts as a direct predictor of 30-year cardiovascular disease risk [36]. Therefore, there is an urgent need on identifying the persistent alterations on cardiovascular physiology associated with ABD to reduce the burden of cardiovascular morbidity in adult life. In the present study we found that arterial blood pressure remains normal in ABD rats during adulthood. Interestingly, both non-invasive echocardiographic analyses and invasive PV loops at PND70 evidenced the presence of marked diastolic dysfunction (Figure 2). Indeed, ABD rats showed significantly higher E/A ratio and a substantial increase in EDPVR. Importantly, cardiac diastolic dysfunction in ABD was not secondary to increases in cardiac afterload since no significant changes were found in blood pressure in the ABD group. A Study of the National Health and Nutrition Examination Survey data (NHANES) showed that young adult men with a repeated history of binge drinking had chronically elevated SBP and total cholesterol [37]. Comparable results were observed in other epidemiological studies assessing cardiorespiratory fitness [38]. This apparent discrepancy with our results is explained by fundamental differences in the study design: whereas the human cohorts evaluated active chronic alcohol consumption from young to adulthood, in our model rats were exposed to a prolonged withdrawal period of abstinence for one month (equivalent to several years in human life [39]). Then, we cannot rule out that acute changes in blood pressure occur in rats during binge-drinking like episodes. Furthermore, whether the changes in cardiac function after prolonged abstinence from binge drinking are or not related to acute hemodynamic alterations during binge-drinking like episodes deserve future investigations.

Our results showed a persistent loss of cardiac compliance in adulthood following ABD. These results extend previous findings reporting increased cardiac stiffness after 6 days of ABD in adolescent rats [33] by showing that cardiac remodeling induced by ABD persists despite a long time of abstinence in rats. Indeed, we found evidence of reactive interstitial fibrosis, tightly coupled with elevated levels of reactive oxygen species (ROS), TNF-α, IL-1β, IL-6 and TGF-β1 mRNA in the adult cardiac tissue. Crucially, this adverse myocardial environment was accompanied by blunted IL-10 mRNA expression. The resulting elevation of the IL-6/IL-10 mRNA ratio serves as an index to quantify the inflammatory balance to the pro-inflammatory state in experimental and human cardiovascular disease [33,40,41]. Mechanistically, this chronic cascade is governed by the sustained activation of the NF-κB pathway, as evidenced by the significant phosphorylation of p65 at Ser536.

Importantly, the long-lasting pro-oxidant/pro-inflammatory milleu resulting from ABD in rats may further contribute to cardiac dysfunction by a feed-forward mechanism that sustained the permanent adverse remodeling of the heart. In fact, unsupervised mathematical modeling of main physiological and molecular variables of this study show that cardiac ROS levels exert a highly predictive value for diastolic dysfunction, autonomic imbalance, cardiac inflammation and fibrosis. However, the exact contribution of oxidative stress/inflammation on the permanent alterations in cardiac function and remodeling after abstinence from ABD remains to be determined.

Autonomic abnormalities have been reported following binge-drinking behaviors. Indeed, chronic binge drinking causes vascular dysfunction [42], associated with increased sympathetic activity (increased LF_HRV_ power) and reduced baroreflex sensitivity in young adults (18–25-years old) [43,44]. Our results showed the establishment of a permanent autonomic imbalance after abstinence from ABD, characterized by higher cardiac sympathetic tone as evidenced by a significant increase in the low-frequency to high-frequency (LF/HF) ratio of HRV. Strikingly, autonomic alterations were accompanied by marked increases in the incidence of spontaneous arrhythmias in adulthood, after a month of ABD withdrawal. Importantly, a systematic review following more than 850,000 patients for 12 years reported that binge drinkers show increased risk of cardiac arrhythmias, stroke and myocardial infarction [45,46]. Moreover, acute binge drinking has been associated with a significant increase in QT dispersion, increased levels of plasma cortisol and systolic arterial pressure [47], supporting the notion that ABD causes acute neural and cardiovascular damage. Our results extend previous results showing the acute effects of ABD on cardiac electrophysiology and cardiovascular physiology but also extend these findings by showing that ABD causes chronic sympathetic hyperactivity and cardiac (mal)adaptations that persist after 1 month of abstinence. The mechanisms linking cardiac sympathoexcitation and tissue remodeling after withdrawal of binge-drinking behavior is completely unknown. However, it has been shown that binge alcohol exposure activates stress kinases (i.e., JNK2), which in turn activates CaMKII leading to the hyperphosphorylation of the cardiac ryanodine receptor (RyR2) resulting in diastolic calcium leak from the sarcoplasmic reticulum, delayed afterdepolarizations and arrhythmias [48,49]. Therefore, it is plausible to hypothesize that heightened sympathetic drive to the heart after abstinence from ABD, increases norepinephrine spillover, adrenergic drive and phosphorylation of intracellular pathways that promote and sustain a pro-inflammatory and pro-arrhythmogenesis niche in the heart which ultimately led to cardiac functional impairments in the adulthood. This novel hypothesis deserves future investigations.

### Study Limitations

While this study provides a comprehensive integrative characterization linking adolescent binge drinking (ABD) to adult cardiac dysfunction, certain experimental scope parameters warrant consideration. Although direct intracellular causal dynamics were not manipulated via targeted genetic knockouts or specific pharmacological inhibition in vivo, our molecular profiling successfully identifies key central transcriptional and signaling nodes driving ABD pathophysiology. Specifically, the chronic pro-inflammatory cascade was firmly supported by the robust activation of the NF-κB pathway supported by p65 phosphorylation and pro-inflammatory cytokine mRNA expression. This triad of collagen deposition driven by TGF-β1, ROS accumulation, and cytokine mRNA overexpression establishes the mechanistic foundation for the mechanical stiffening (elevated EDPVR) and electrical instability observed in vivo. Consequently, our findings establish the central pathophysiological axis:

ABD→ NF-κB ↔ ROS ↔ TGF-β1/Collagen Deposition → Diastolic Dysfunction.

This structural and molecular framework offers a well-defined baseline to guide future targeted interventional therapeutics and mechanistic causality trials.

Secondly, the experimental cohort was restricted to male rats to control the potential confounding effects of the estrous cycle on ABD pathophysiology [50], given the well-documented sex dimorphisms in cardiovascular and redox physiology [51,52]. Despite available evidence, data regarding binge drinking show no significant differences in echocardiographic parameters and arrhythmia susceptibility [48] and similar results in autonomic imbalance via HRV among males and females [50]. No sex-dysmorphic studies have been conducted yet regarding ROS and pro-inflammatory mediator mRNA expression in adult heart subjected to binge drinking in adolescence.

Thirdly, while our study characterized the long-term myocardial-specific pro-inflammatory and pro-oxidant footprint at PND 70, circulating systemic biomarkers of oxidative stress and inflammation were not longitudinally evaluated in blood samples during the active binge-drinking protocol (PND 38). Although the presence of acute systemic physiological stress during adolescent exposure was confirmed by a transient elevation in blood pressure at PND 38, which subsequently normalized by PND 70, the lack of acute blood chemistry data restricts our ability to correlate early circulating systemic inflammatory kinetics with the magnitude of chronic local myocardial remodeling

## 5. Conclusions

Present results identify ABD as a critical insult that surpasses the acute toxicological effects, establishing a state of chronic redox-driven fibrotic phenotype in the heart that persists until adult life, even following a long withdrawal period from late adolescence to early adulthood. Unlike adult-onset alcohol exposure, ABD occurs during a period of cardiovascular maturation, when cardiomyocytes are especially sensitive to oxidative and inflammatory damage [53]. Present results evidenced high levels of reactive oxygen species, TNF-α and IL-1β in the heart of ABD rats. This was accompanied by cardiac fibrosis and arrhythmogenesis being the outcome of an increase in left ventricle stiffness and cardiac diastolic dysfunction.

In summary, our findings support that ABD triggers the persistent activation of pro-oxidative and pro-inflammatory pathways in the heart which may ultimately lead to adverse tissue remodeling and cardiac dysfunction even after long-time alcohol withdrawal.

## Data Availability

The original contributions presented in this study are included in the article and Supplementary Materials.

## Supplementary Materials

The following supporting information can be downloaded at https://www.mdpi.com/article/10.3390/antiox15101360/s1, Table S1: Comparative Electrophysiological and Arrhythmic Parameters; Table S2: Multiple variable correlations. Table S3: Principal component regression. Table S4: Mean 2^−ΔΔCt^ values of RT-qPCR quantifications. Figure S1: mRNA expression of pro/anti-inflammatory and profibrotic genes in control and ABD rats. Figure S2: Multivariate correlation results. Supplementary File S1: original images for microscopy analyses.

## Abbreviations

The following abbreviations are used in this manuscript:

ABD	Adolescent binge drinking
ROS	Reactive oxygen species
IL-1β	Interleukin-1β
TNF-α	Tumor necrosis factor-alpha
PV-loops	Pressure-volume loops
PND	Postnatal day
HRV	Heart rate variability
DHE	Dihydroethidium
EKG	Electrocardiogram
LVEDV	Left-ventricular end-diastolic volume
LVESV	Left-ventricular end-systolic volume
SV	Stroke volume
CO	Cardiac output
FS	Fractional shortening
EF	Ejection fraction
LV	Left Ventricular
ESPVR	End-systolic pressure-volume relationship
EDPVR	End-diastolic pressure-volume relationship
LF_HRV_	Low-frequency component of heart rate variability
HF_HRV_	High-frequency component of heart rate variability
BP	Blood pressure
SBP	Systolic blood pressure
DBP	Diastolic blood pressure
HR	Heart rate
PSD	Power spectral density
PVC	Premature ventricular contraction
VT	Ventricular tachycardia
VF	Ventricular fibrillation
IL-6	Interleukin-6
IL-10	Interleukin-6
TGF-β1	Transforming growth factor-β1
NF-κB	Nuclear factor kappa B
SOD2	Superoxide Dismutase 2
NOX2	NADPH Oxidase 2
