# Supplementary material for: Binge Drinking During Adolescence Induces Long-Lasting Oxidative Stress and Inflammation in the Heart and Promotes Cardiac Dysfunction in Rats
Source: Antioxidants (Basel). 2026 Oct 21;15(10):1360. doi: 10.3390/antiox15101360 (PMC13701238; doi:10.3390/antiox15101360)
Supplement: Supplementary file 1 [file antioxidants-15-01360-s001.zip › Supplementary Figure S1.pdf]

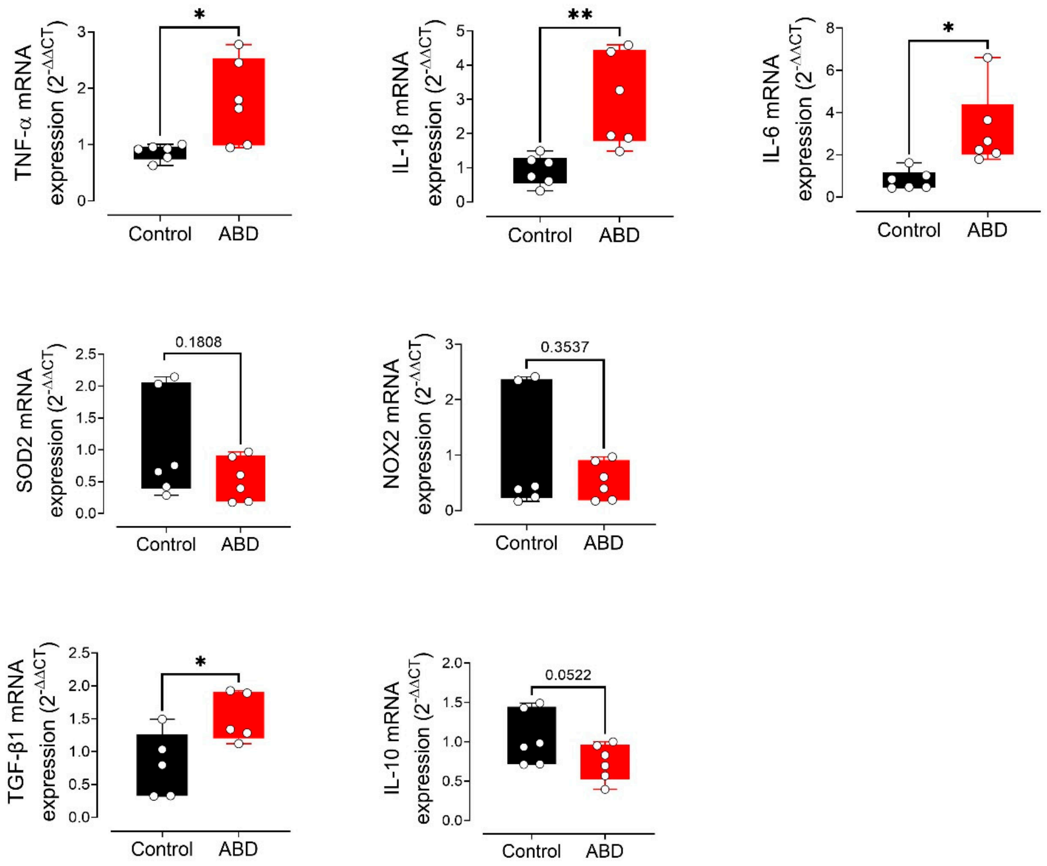

Supplementary Figure S1. mRNA expression of pro/anti-inflammatory and profibrotic genes in control and ABD rats. RT-qPCR results showing the myocardial mRNA expression of 6 control and 6 ABD rats. Unpaired t test with Welch's correction. *: $p < 0.05$; **: $p < 0.01$
