# Supplementary material for: Binge Drinking During Adolescence Induces Long-Lasting Oxidative Stress and Inflammation in the Heart and Promotes Cardiac Dysfunction in Rats
Source: Antioxidants (Basel). 2026 Oct 21;15(10):1360. doi: 10.3390/antiox15101360 (PMC13701238; doi:10.3390/antiox15101360)
Supplement: Supplementary file 1 [file antioxidants-15-01360-s001.zip › Supplementary Figure S2.pdf]

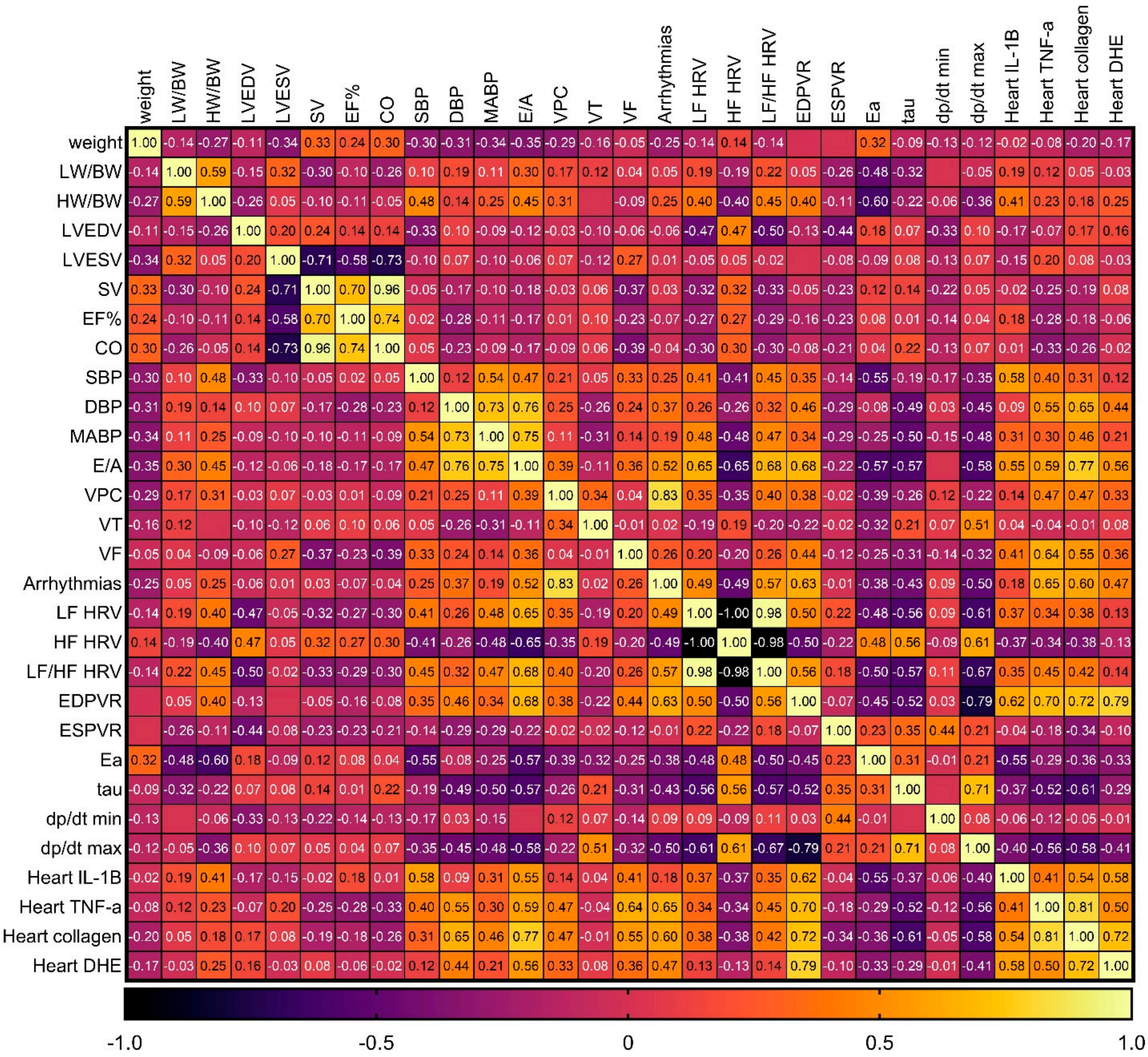


**Supplementary Figure S2. Multivariate correlation results**. Pearson-r correlation heatmap in a 30 x 30 matrix including 24 subjects LW: lung weight; HW: heart weight; BW: body weight; LVEDV: left-ventricular end-diastolic volume; LVESV: left-ventricular end-systolic volume; SV: stroke volume; CO: cardiac output; FS: fractional shortening; EF: ejection fraction; SBP: systolic blood pressure; DBP: diastolic blood pressure; MABP: mean arterial pressure; HR: heart rate; HRV: heart rate variability; LFHRV: low-frequency component of heart rate variability; HFHRV: high-frequency component of heart rate variability; ESPVR: End-systolic pressure–volume relationship; EDPVR: end-diastolic pressure–volume relationship; PVC: premature ventricular contraction; VT: ventricular tachycardia; VF: ventricular fibrillation; IL-1β: interleukin-1β; TNF-α: tumor necrosis factor-alpha; DHE: dihydroethidium.
