# Supplementary material for: Binge Drinking During Adolescence Induces Long-Lasting Oxidative Stress and Inflammation in the Heart and Promotes Cardiac Dysfunction in Rats
Source: Antioxidants (Basel). 2026 Oct 21;15(10):1360. doi: 10.3390/antiox15101360 (PMC13701238; doi:10.3390/antiox15101360)
Supplement: Supplementary file 1 [file antioxidants-15-01360-s001.zip › Supplementary Table S1.pdf]

**Supplementary Table S1. Comparative Electrophysiological and Arrhythmic Parameters**

| **Variable** | **Control (n = 6)** | **ABD (n = 6)** | **p-value** |
|---|---|---|---|
| RR Interval (s) | 0.157 ± 0.007 | 0.154 ± 0.009 | 0.812 |
| PR Interval (s) | 0.049 ± 0.005 | 0.045 ± 0.004 | 0.534 |
| QRS Interval (s) | 0.031 ± 0.004 | 0.068 ± 0.026 | 0.178 |
| QTc (s) | 0.202 ± 0.013 | 0.151 ± 0.086 | 0.570 |
| Total PVCs (events) | 11.00 ± 5.09 | 16.50 ± 11.23 | 0.672 |
| Total VT (events) | 3.50 ± 3.50 | 4.00 ± 2.65 | 0.913 |
| Total VF (events) | 0.17 ± 0.17 | 5.25 ± 2.40 | 0.062 |
| Total VF Time (s) | 0.03 ± 0.03 | 1.78 ± 1.15 | **0.03*** |
| G-Score | 2.39 ± 0.59 | 4.80 ± 0.81 | **0.038*** |

Arrhythmic events were counted and classified in 20-minute segments and then scored in the G-Score system. Values presented as mean ± SEM. p-values derived via independent t-test. *$p<0.05$
