# Supplementary material for: Binge Drinking During Adolescence Induces Long-Lasting Oxidative Stress and Inflammation in the Heart and Promotes Cardiac Dysfunction in Rats
Source: Antioxidants (Basel). 2026 Oct 21;15(10):1360. doi: 10.3390/antiox15101360 (PMC13701238; doi:10.3390/antiox15101360)
Supplement: Supplementary file 1 [file antioxidants-15-01360-s001.zip › Supplementary Table S2.pdf]

Supplementary Table S2. Multiple variable correlations.

	weight	LW/BW	HW/BW	LVEDV	LVESV	SV	EF%	CO	SBP	DBP	MABP	E/A	VPC	VT	VF	Arrhyth-mias	LF HRV	HF HRV	LF/HF HRV	EDPVR	ESPVR	Ea	tau	dp/dt min	dp/dt max	Heart IL-1B	Heart TNF-a	Heart collagen	Heart DHE
weight	1.000	-0.143	-0.270	-0.109	-0.336	0.329	0.239	0.304	-0.298	-0.312	-0.340	-0.347	-0.294	-0.161	-0.053	-0.251	-0.140	0.140	-0.137	-0.005	-0.001	0.323	-0.090	-0.125	-0.120	-0.020	-0.084	-0.201	-0.171
LW/BW	-0.143	1.000	0.590	-0.147	0.318	-0.298	-0.100	-0.261	0.104	0.188	0.109	0.304	0.173	0.120	0.039	0.046	0.192	-0.192	0.222	0.048	-0.261	-0.484	-0.323	0.001	-0.050	0.192	0.120	0.053	-0.029
HW/BW	-0.270	0.590	1.000	-0.259	0.049	-0.102	-0.106	-0.050	0.483	0.143	0.249	0.445	0.306	0.003	-0.091	0.255	0.403	-0.403	0.446	0.405	-0.113	-0.600	-0.223	-0.064	-0.357	0.412	0.230	0.184	0.249
LVEDV	-0.109	-0.147	-0.259	1.000	0.198	0.244	0.137	0.136	-0.333	0.098	-0.086	-0.118	-0.025	-0.101	-0.056	-0.062	-0.465	0.465	-0.502	-0.128	-0.438	0.176	0.075	-0.329	0.104	-0.170	-0.070	0.169	0.159
LVESV	-0.336	0.318	0.049	0.198	1.000	-0.705	-0.579	-0.734	-0.103	0.066	-0.101	-0.065	0.071	-0.122	0.274	0.014	-0.051	0.051	-0.019	-0.002	-0.076	-0.093	0.079	-0.129	0.070	-0.154	0.204	0.083	-0.031
SV	0.329	-0.298	-0.102	0.244	-0.705	1.000	0.700	0.964	-0.047	-0.168	-0.102	-0.183	-0.029	0.062	-0.374	0.032	-0.323	0.323	-0.334	-0.049	-0.228	0.123	0.141	-0.217	0.051	-0.023	-0.253	-0.187	0.077
EF%	0.239	-0.100	-0.106	0.137	-0.579	0.700	1.000	0.737	0.022	-0.282	-0.114	-0.174	0.014	0.096	-0.231	-0.072	-0.267	0.267	-0.292	-0.157	-0.232	0.078	0.007	-0.137	0.043	0.182	-0.284	-0.175	-0.062
CO	0.304	-0.261	-0.050	0.136	-0.734	0.964	0.737	1.000	0.055	-0.232	-0.093	-0.166	-0.090	0.056	-0.391	-0.041	-0.295	0.295	-0.299	-0.082	-0.208	0.038	0.216	-0.129	0.071	0.013	-0.331	-0.265	-0.017
SBP	-0.298	0.104	0.483	-0.333	-0.103	-0.047	0.022	0.055	1.000	0.117	0.543	0.470	0.209	0.052	0.329	0.254	0.410	-0.410	0.454	0.347	-0.143	-0.548	-0.189	-0.173	-0.352	0.580	0.401	0.307	0.121
DBP	-0.312	0.188	0.143	0.098	0.066	-0.168	-0.282	-0.232	0.117	1.000	0.729	0.759	0.246	-0.261	0.241	0.375	0.262	-0.262	0.315	0.463	-0.293	-0.078	-0.487	0.027	-0.453	0.092	0.546	0.653	0.442
MABP	-0.340	0.109	0.249	-0.086	-0.101	-0.102	-0.114	-0.093	0.543	0.729	1.000	0.746	0.113	-0.308	0.141	0.192	0.481	-0.481	0.474	0.342	-0.292	-0.246	-0.496	-0.146	-0.475	0.313	0.297	0.455	0.211
E/A	-0.347	0.304	0.445	-0.118	-0.065	-0.183	-0.174	-0.166	0.470	0.759	0.746	1.000	0.390	-0.106	0.360	0.523	0.651	-0.651	0.677	0.682	-0.220	-0.570	-0.574	0.001	-0.584	0.548	0.590	0.771	0.556
VPC	-0.294	0.173	0.306	-0.025	0.071	-0.029	0.014	-0.090	0.209	0.246	0.113	0.390	1.000	0.344	0.042	0.830	0.354	-0.354	0.405	0.380	-0.016	-0.386	-0.260	0.115	-0.225	0.141	0.466	0.466	0.330
VT	-0.161	0.120	0.003	-0.101	-0.122	0.062	0.096	0.056	0.052	-0.261	-0.308	-0.106	0.344	1.000	-0.008	0.024	-0.190	0.190	-0.196	-0.220	-0.022	-0.323	0.209	0.069	0.510	0.041	-0.043	-0.008	0.078
VF	-0.053	0.039	-0.091	-0.056	0.274	-0.374	-0.231	-0.391	0.329	0.241	0.141	0.360	0.042	-0.008	1.000	0.260	0.205	-0.205	0.258	0.441	-0.119	-0.248	-0.312	-0.138	-0.318	0.413	0.637	0.545	0.357
Arrhythmias	-0.251	0.046	0.255	-0.062	0.014	0.032	-0.072	-0.041	0.254	0.375	0.192	0.523	0.830	0.024	0.260	1.000	0.492	-0.492	0.566	0.632	-0.009	-0.382	-0.431	0.089	-0.497	0.183	0.652	0.597	0.471
LF HRV	-0.140	0.192	0.403	-0.465	-0.051	-0.323	-0.267	-0.295	0.410	0.262	0.481	0.651	0.354	-0.190	0.205	0.492	1.000	-1.000	0.981	0.496	0.225	-0.477	-0.562	0.095	-0.615	0.371	0.344	0.377	0.135
HF HRV	0.140	-0.192	-0.403	0.465	0.051	0.323	0.267	0.295	-0.410	-0.262	-0.481	-0.651	-0.354	0.190	-0.205	-0.492	-1.000	1.000	-0.981	-0.496	-0.225	0.477	0.562	-0.095	0.615	-0.371	-0.344	-0.377	-0.135
LF/HF HRV	-0.137	0.222	0.446	-0.502	-0.019	-0.334	-0.292	-0.299	0.454	0.315	0.474	0.677	0.405	-0.196	0.258	0.566	0.981	-0.981	1.000	0.558	0.181	-0.501	-0.573	0.111	-0.665	0.346	0.454	0.415	0.141
EDPVR	-0.005	0.048	0.405	-0.128	-0.002	-0.049	-0.157	-0.082	0.347	0.463	0.342	0.682	0.380	-0.220	0.441	0.632	0.496	-0.496	0.558	1.000	-0.069	-0.451	-0.523	0.029	-0.788	0.621	0.698	0.724	0.792
ESPVR	-0.001	-0.261	-0.113	-0.438	-0.076	-0.228	-0.232	-0.208	-0.143	-0.293	-0.292	-0.220	-0.016	-0.022	-0.119	-0.009	0.225	-0.225	0.181	-0.069	1.000	0.228	0.352	0.443	0.207	-0.039	-0.175	-0.344	-0.098
Ea	0.323	-0.484	-0.600	0.176	-0.093	0.123	0.078	0.038	-0.548	-0.078	-0.246	-0.570	-0.386	-0.323	-0.248	-0.382	-0.477	0.477	-0.501	-0.451	0.228	1.000	0.307	-0.011	0.210	-0.551	-0.293	-0.362	-0.326
tau	-0.090	-0.323	-0.223	0.075	0.079	0.141	0.007	0.216	-0.189	-0.487	-0.496	-0.574	-0.260	0.209	-0.312	-0.431	-0.562	0.562	-0.573	-0.523	0.352	0.307	1.000	-0.001	0.714	-0.373	-0.520	-0.611	-0.288
dp/dt min	-0.125	0.001	-0.064	-0.329	-0.129	-0.217	-0.137	-0.129	-0.173	0.027	-0.146	0.001	0.115	0.069	-0.138	0.089	0.095	-0.095	0.111	0.029	0.443	-0.011	-0.001	1.000	0.076	-0.065	-0.123	-0.054	-0.012
dp/dt max	-0.120	-0.050	-0.357	0.104	0.070	0.051	0.043	0.071	-0.352	-0.453	-0.475	-0.584	-0.225	0.510	-0.318	-0.497	-0.615	0.615	-0.665	-0.788	0.207	0.210	0.714	0.076	1.000	-0.398	-0.557	-0.581	-0.409
Heart IL-1B	-0.020	0.192	0.412	-0.170	-0.154	-0.023	0.182	0.013	0.580	0.092	0.313	0.548	0.141	0.041	0.413	0.183	0.371	-0.371	0.346	0.621	-0.039	-0.551	-0.373	-0.065	-0.398	1.000	0.413	0.538	0.583
Heart TNF-a	-0.084	0.120	0.230	-0.070	0.204	-0.253	-0.284	-0.331	0.401	0.546	0.297	0.590	0.466	-0.043	0.637	0.652	0.344	-0.344	0.454	0.698	-0.175	-0.293	-0.520	-0.123	-0.557	0.413	1.000	0.814	0.504
Heart collagen	-0.201	0.053	0.184	0.169	0.083	-0.187	-0.175	-0.265	0.307	0.653	0.455	0.771	0.466	-0.008	0.545	0.597	0.377	-0.377	0.415	0.724	-0.344	-0.362	-0.611	-0.054	-0.581	0.538	0.814	1.000	0.716
Heart DHE	-0.171	-0.029	0.249	0.159	-0.031	0.077	-0.062	-0.017	0.121	0.442	0.211	0.556	0.330	0.078	0.357	0.471	0.135	-0.135	0.141	0.792	-0.098	-0.326	-0.288	-0.012	-0.409	0.583	0.504	0.716	1.000

LW: lung weight; HW: heart weight; BW: body weight; LVEDV: left-ventricular end-diastolic volume; LVESV: left-ventricular end-systolic volume; SV: stroke volume; CO: cardiac output; FS: fractional shortening; EF: ejection fraction; SBP: systolic blood pressure; DBP: diastolic blood pressure; MABP: mean arterial pressure; HR: heart rate; HRV: heart rate variability; LFHRV: low-frequency component of heart rate variability; HFHRV: high-frequency component of heart rate variability; ESPVR: end-systolic pressure–volume relationship; EDPVR: end-diastolic pressure–volume relationship; PVC: premature ventricular contraction; VT: ventricular tachycardia; VF: ventricular fibrillation; IL-1β: interleukin-1β; TNF-α: tumor necrosis factor-alpha; DHE: dihydroethidium.

P-value summary of multiple correlations

	weight	LW/BW	HW/BW	LVEDV	LVESV	SV	EF%	CO	SBP	DBP	MABP	E/A	VPC	VT	VF	Arrhythmias	LF HRV	HF HRV	LF/HF HRV	EDPVR	ESPVR	Ea	tau	dp/dt min	dp/dt max	Heart IL-1B	Heart TNF-a	Heart collagen	Heart DHE
weight		0.50520751	0.20220898	0.61309573	0.10859632	0.11594284	0.26111256	0.14875278	0.15698905	0.13737169	0.10447503	0.09702965	0.16273818	0.45154678	0.80635712	0.23721725	0.51260896	0.51260896	0.52466897	0.98156323	0.99630579	0.12323526	0.67748815	0.56012039	0.57599392	0.92486729	0.69738878	0.34599438	0.42400346
LW/BW	0.50520751		0.0024142	0.49254301	0.12960615	0.15655023	0.6431474	0.21730787	0.62792224	0.3795351	0.61236056	0.148049	0.41858065	0.57767436	0.85488851	0.83171797	0.36980364	0.36980364	0.29669477	0.82250206	0.21764991	0.01651606	0.12391969	0.99678709	0.81547543	0.36857466	0.57766286	0.80440074	0.89298596
HW/BW	0.20220898	0.0024142		0.22120762	0.81903427	0.636643	0.62196622	0.81559223	0.01686777	0.50377707	0.24010066	0.02920392	0.14539087	0.98754531	0.67407839	0.22955172	0.05116311	0.05116311	0.02874369	0.04992676	0.59890005	0.00192627	0.29401949	0.76806571	0.08690288	0.0456928	0.27982683	0.3900232	0.24114969
LVEDV	0.61309573	0.49254301	0.22120762		0.35253397	0.25029282	0.52431239	0.52540949	0.11137131	0.64815355	0.68902904	0.58327643	0.90587397	0.63715952	0.79479582	0.77327035	0.02195949	0.02195949	0.0124752	0.55030875	0.03240046	0.41186877	0.72806988	0.11611172	0.62857885	0.42744203	0.74599652	0.43123252	0.45940976
LVESV	0.10859632	0.12960615	0.81903427	0.35253397		0.00011781	0.00302264	4.4108E-05	0.63294734	0.76007829	0.63963388	0.76382877	0.7414356	0.56879911	0.19510595	0.94810051	0.81248968	0.81248968	0.92980065	0.99193988	0.72484829	0.66519821	0.7122887	0.54781962	0.74679173	0.47346332	0.34014506	0.70148298	0.88536802
SV	0.11594284	0.15655023	0.636643	0.25029282	0.00011781		0.00013905	3.4319E-14	0.82566195	0.43150047	0.63474515	0.39246992	0.89368225	0.77371411	0.0719847	0.88340901	0.12316234	0.12316234	0.11017613	0.82148018	0.28434224	0.566027	0.50993854	0.30907524	0.81434932	0.91595439	0.23213294	0.38098071	0.72099761
EF%	0.26111256	0.6431474	0.62196622	0.52431239	0.00302264	0.00013905		3.9453E-05	0.91794466	0.18137649	0.59517683	0.41695838	0.94773765	0.65420894	0.27741566	0.7380351	0.20725119	0.20725119	0.16669819	0.4637936	0.27462309	0.7160397	0.97353209	0.52317345	0.84057789	0.3949221	0.17867628	0.41247696	0.77209706
CO	0.14875278	0.21730787	0.81559223	0.52540949	4.4108E-05	3.4319E-14	3.9453E-05		0.79866051	0.27603864	0.66673697	0.43698064	0.67687033	0.79353479	0.05905971	0.84882222	0.16116652	0.16116652	0.1560819	0.7026809	0.32899195	0.85951902	0.3115138	0.54650326	0.74240868	0.95080555	0.11359844	0.21086701	0.93808163
SBP	0.15698905	0.62792224	0.01686777	0.11137131	0.63294734	0.82566195	0.91794466	0.79866051		0.58628387	0.00607165	0.02039182	0.32793141	0.81083405	0.11693679	0.23108918	0.04669468	0.04669468	0.02580787	0.09707564	0.50370959	0.00554294	0.37747721	0.41879638	0.0917499	0.00299893	0.05211243	0.14410353	0.57186045
DBP	0.13737169	0.3795351	0.50377707	0.64815355	0.76007829	0.43150047	0.18137649	0.27603864	0.58628387		5.3796E-05	1.7293E-05	0.24602834	0.21810679	0.25576547	0.07127126	0.21570884	0.21570884	0.13336308	0.02282736	0.16403526	0.7187304	0.01588529	0.89974515	0.02618483	0.6689395	0.00581377	0.00053505	0.03053968
MABP	0.10447503	0.61236056	0.24010066	0.68902904	0.63963388	0.63474515	0.59517683	0.66673697	0.00607165	5.3796E-05		2.8873E-05	0.59830711	0.14373555	0.51153118	0.36803829	0.01733853	0.01733853	0.01918426	0.10208165	0.16560451	0.24719585	0.01367813	0.49686078	0.01894479	0.13640514	0.15909754	0.02546624	0.32225506
E/A	0.09702965	0.148049	0.02920392	0.58327643	0.76382877	0.39246992	0.41695838	0.43698064	0.02039182	1.7293E-05	2.8873E-05		0.05981736	0.62170567	0.08434117	0.0087209	0.00057192	0.00057192	0.00027741	0.00024451	0.30141432	0.00361227	0.00334336	0.99559178	0.00270955	0.00558496	0.00239199	1.0591E-05	0.00481417
VPC	0.16273818	0.41858065	0.14539087	0.90587397	0.7414356	0.89368225	0.94773765	0.67687033	0.32793141	0.24602834	0.59830711	0.05981736		0.10011827	0.84398143	5.1754E-07	0.08962227	0.08962227	0.04981267	0.06737453	0.93929118	0.06233278	0.2206615	0.59191375	0.2906396	0.5122757	0.02171315	0.02162443	0.11586559
VT	0.45154678	0.57767436	0.98754531	0.63715952	0.56879911	0.77371411	0.65420894	0.79353479	0.81083405	0.21810679	0.14373555	0.62170567	0.10011827		0.9695927	0.91160098	0.37320453	0.37320453	0.35805076	0.30060139	0.91734555	0.12372313	0.32587266	0.74875771	0.01093801	0.84879987	0.84086608	0.97089159	0.7181211
VF	0.80635712	0.85488851	0.67407839	0.79479582	0.19510595	0.0719847	0.27741566	0.05905971	0.11693679	0.25576547	0.51153118	0.08434117	0.84398143	0.9695927		0.22072597	0.33715808	0.33715808	0.22352332	0.03120067	0.5809549	0.24232352	0.13790985	0.52028703	0.13050347	0.04514388	0.00081712	0.00586143	0.0866331
Arrhythmias	0.23721725	0.83171797	0.22955172	0.77327035	0.94810051	0.88340901	0.7380351	0.84882222	0.23108918	0.07127126	0.36803829	0.0087209	5.1754E-07	0.91160098	0.22072597		0.01460473	0.01460473	0.0039546	0.00091532	0.96749379	0.06520764	0.03546402	0.6805399	0.01356376	0.39234828	0.00055903	0.00205301	0.020282
LF HRV	0.51260896	0.36980364	0.05116311	0.02195949	0.81248968	0.12316234	0.20725119	0.16116652	0.04669468	0.21570884	0.01733853	0.00057192	0.08962227	0.37320453	0.33715808	0.01460473		0	3.6131E-17	0.01360535	0.2909474	0.01854619	0.00429127	0.65996543	0.00139004	0.07445075	0.10027068	0.06936719	0.53005071
HF HRV	0.51260896	0.36980364	0.05116311	0.02195949	0.81248968	0.12316234	0.20725119	0.16116652	0.04669468	0.21570884	0.01733853	0.00057192	0.08962227	0.37320453	0.33715808	0.01460473	0		3.6131E-17	0.01360535	0.2909474	0.01854619	0.00429127	0.65996543	0.00139004	0.07445075	0.10027068	0.06936719	0.53005071
LF/HF	0.52466897	0.29669477	0.02874369	0.0124752	0.92980065	0.11017613	0.16669819	0.1560819	0.02580787	0.13336308	0.01918426	0.00027741	0.04981267	0.35805076	0.22352332	0.0039546	3.6131E-17	3.6131E-17		0.0045756	0.39693067	0.01259306	0.00340841	0.60460279	0.00039202	0.09731754	0.02596058	0.04350466	0.51093096
EDPVR	0.98156323	0.82250206	0.04992676	0.55030875	0.99193988	0.82148018	0.4637936	0.7026809	0.09707564	0.02282736	0.10208165	0.00024451	0.06737453	0.30060139	0.03120067	0.00091532	0.01360535	0.01360535	0.0045756		0.74982045	0.02714637	0.00879127	0.89376838	4.7475E-06	0.00119223	0.00014735	6.2421E-05	3.9756E-06
ESPVR	0.99630579	0.21764991	0.59890005	0.03240046	0.72484829	0.28434224	0.27462309	0.32899195	0.50370959	0.16403526	0.16560451	0.30141432	0.93929118	0.91734555	0.5809549	0.96749379	0.2909474	0.2909474	0.39693067	0.74982045		0.28500923	0.09208318	0.03022365	0.33182534	0.85719816	0.41260959	0.09992785	0.65024937
Ea	0.12323526	0.01651606	0.00192627	0.41186877	0.66519821	0.566027	0.7160397	0.85951902	0.00554294	0.7187304	0.24719585	0.00361227	0.06233278	0.12372313	0.24232352	0.06520764	0.01854619	0.01854619	0.01259306	0.02714637	0.28500923		0.14486917	0.96053899	0.32489071	0.00529535	0.16447854	0.08202725	0.12033176
tau	0.67748815	0.12391969	0.29401949	0.72806988	0.7122887	0.50993854	0.97353209	0.3115138	0.37747721	0.01588529	0.01367813	0.00334336	0.2206615	0.32587266	0.13790985	0.03546402	0.00429127	0.00429127	0.00340841	0.00879127	0.09208318	0.14486917		0.99739726	9.0049E-05	0.07279827	0.0092371	0.00150257	0.1719837
dp/dt min	0.56012039	0.99678709	0.76806571	0.11611172	0.54781962	0.30907524	0.52317345	0.54650326	0.41879638	0.89974515	0.49686078	0.99559178	0.59191375	0.74875771	0.52028703	0.6805399	0.65996543	0.65996543	0.60460279	0.89376838	0.03022365	0.96053899	0.99739726		0.72354433	0.7637036	0.56785581	0.80136494	0.9562716
dp/dt max	0.57599392	0.81547543	0.08690288	0.62857885	0.74679173	0.81434932	0.84057789	0.74240868	0.0917499	0.02618483	0.01894479	0.00270955	0.2906396	0.01093801	0.13050347	0.01356376	0.00139004	0.00139004	0.00039202	4.7475E-06	0.33182534	0.32489071	9.0049E-05	0.72354433		0.05396501	0.00466064	0.00288801	0.04740264
Heart IL-1B	0.92486729	0.36857466	0.0456928	0.42744203	0.47346332	0.91595439	0.3949221	0.95080555	0.00299893	0.6689395	0.13640514	0.00558496	0.5122757	0.84879987	0.04514388	0.39234828	0.07445075	0.07445075	0.09731754	0.00119223	0.85719816	0.00529535	0.07279827	0.7637036	0.05396501		0.04484359	0.00668914	0.00277119
TNF-a	0.69738878	0.57766286	0.27982683	0.74599652	0.34014506	0.23213294	0.17867628	0.11359844	0.05211243	0.00581377	0.15909754	0.00239199	0.02171315	0.84086608	0.00081712	0.00055903	0.10027068	0.10027068	0.02596058	0.00014735	0.41260959	0.16447854	0.0092371	0.56785581	0.00466064	0.04484359		1.2937E-06	0.01202756
collagen	0.34599438	0.80440074	0.3900232	0.43123252	0.70148298	0.38098071	0.41247696	0.21086701	0.14410353	0.00053505	0.02546624	1.0591E-05	0.02162443	0.97089159	0.00586143	0.00205301	0.06936719	0.06936719	0.04350466	6.2421E-05	0.09992785	0.08202725	0.00150257	0.80136494	0.00288801	0.00668914	1.2937E-06		8.3884E-05
Heart DHE	0.42400346	0.89298596	0.24114969	0.45940976	0.88536802	0.72099761	0.77209706	0.93808163	0.57186045	0.03053968	0.32225506	0.00481417	0.11586559	0.7181211	0.0866331	0.020282	0.53005071	0.53005071	0.51093096	3.9756E-06	0.65024937	0.12033176	0.1719837	0.9562716	0.04740264	0.00277119	0.01202756	8.3884E-05	

LW: lung weight; HW: heart weight; BW: body weight; LVEDV: Left-ventricular end-diastolic volume; LVESV: Left-ventricular end-systolic volume; SV: Stroke Volume; CO: Cardiac Output; FS: Fractional Shortening; EF: Ejection Fraction; SBP: Systolic Blood Pressure; DBP: Diastolic Blood Pressure; MABP: Mean arterial pressure; HR: Heart Rate; HRV: Heart Rate Variability; LFHRV: Low Frequency Component of Heart Rate Variability; HFHRV: High Frequency Component of Heart Rate Variability; ESPVR: End-systolic pressure-volume relationship; EDPVR: End-diastolic pressure-volume relationship; PVC: Premature ventricular contraction; VT: Ventricular tachycardia; VF: Ventricular fibrillation; IL-1β: Interleukin-1β; TNF-α: tumor necrosis factor-alpha; DHE: Dihydroethidium.
