# Supplementary material for: Binge Drinking During Adolescence Induces Long-Lasting Oxidative Stress and Inflammation in the Heart and Promotes Cardiac Dysfunction in Rats
Source: Antioxidants (Basel). 2026 Oct 21;15(10):1360. doi: 10.3390/antiox15101360 (PMC13701238; doi:10.3390/antiox15101360)
Supplement: Supplementary file 1 [file antioxidants-15-01360-s001.zip › Supplementary Table S3.pdf]

Supplementary Table S3. Principal component regression.

Dependent variable	AC : Heart DHE						
Regression type	Least squares						
Model							
**Analysis of Variance**	**SS**	**DF**	**MS**	**F (DFn. DFd)**	**P value**		
Regression	4.954E+09	18	275222451	F (18. 5) = 4.382	P=0.0547		
Residual	314061336	5	62812267				
Total	5.268E+09	23					
**Parameter estimates**	**Variable**	**Estimate**	**Standard error**	**95% CI (asymptotic)**	**|t|**	**P value**	**P value summary**
β0	Intercept	-94852	157687	-500198 to 310495	0.6015	0.5737	ns
β1	weight	187.2	187.3	-294.4 to 668.8	0.9992	0.3636	ns
β2	LW/BW	-1503	476	-2727 to -279.4	3.158	0.0252	*
β3	HW/BW	-287.7	130.7	-623.6 to 48.17	2,202	0.0789	ns
β4	LVEDV	-0.02931	99.15	-254.9 to 254.9	0.0002956	0.9998	ns
β5	LVESV	-77.94	71.06	-260.6 to 104.7	1,097	0.3227	ns
β6	SV	986.7	241.7	365.4 to 1608	4,083	0.0095	**
β7	EF%	2004	737.6	107.8 to 3900	2,717	0.0419	*
β8	CO	-3,254	0.6584	-4.947 to -1.562	4,943	0.0043	**
β9	SBP	-469.2	453.1	-1634 to 695.5	1.036	0.3478	ns
β10	DBP	372	548.3	-1037 to 1781	0.6784	0.5276	ns
β11	MABP	1514	1095	-1300 to 4329	1.383	0.2252	ns
β12	E/A	130.7	34.3	42.49 to 218.8	3,809	0.0125	*
β13	VPC	-713.1	376.6	-1681 to 254.9	1.894	0.1168	ns
β14	VT	714.6	741.1	-1190 to 2620	0.9642	0.3792	ns

β15	VF	-758.2	987.1	-3296 to 1779	0.7682	0.4771	ns
β16	Arrhythmias	390.8	228	-195.3 to 976.9	1,714	0.1472	ns
β17	LF HRV	-578.7	325.9	-1417 to 259.1	1.776	0.136	ns
β18	HF HRV	578.7	325.9	-259.1 to 1417	1,776	0.136	ns
β19	LF/HF HRV	-142.3	38.52	-241.3 to -43.30	3,695	0.0141	*
β20	EDPVR	21.41	4,635	9.492 to 33.32	4,618	0.0057	**
β21	ESPVR	165.1	39.76	62.84 to 267.3	4,151	0.0089	**
β22	Ea	-69.4	16.11	-110.8 to -28.00	4,309	0.0077	**
β23	tau	4289	851	2101 to 6476	5,040	0.004	**
β24	dp/dt min	4,684	2,523	-1.803 to 11.17	1,856	0.1226	ns
β25	dp/dt max	-0.002922	0.0007437	-0.004834 to -0.001011	3,930	0.0111	*
β26	Heart IL-1B	2556	623.9	952.1 to 4160	4,097	0.0094	**
β27	Heart TNF-a	79.65	174.5	-368.8 to 528.1	0.4566	0.6671	ns
β28	Heart collagen	860.9	241.1	241.2 to 1481	3,571	0.016	*

**Goodness of Fit**	
Degrees of Freedom	5
R squared	0.9404
Sum of Squares	314061336
Sy.x	7925

**Data summary**	
Total number of variables	28
Total number of components	23
Component selection method	PCs with eigenvalues greater than 1.00
Number of selected components	18

Rows in table	24
Rows skipped (missing data)	0
Rows analyzed (# cases)	24

LW: lung weight; HW: heart weight; BW: body weight; LVEDV: left-ventricular end-diastolic volume; LVESV: left-ventricular end-systolic volume; SV: stroke volume; CO: cardiac output; FS: fractional shortening; EF: ejection fraction; SBP: systolic blood pressure; DBP: diastolic blood pressure; MABP: mean arterial pressure; HR: heart rate; HRV: heart rate variability; LFHRV: low-frequency component of heart rate variability; HFHRV: high-frequency component of heart rate variability; ESPVR: end-systolic pressure–volume relationship; EDPVR: end-diastolic pressure–volume relationship; PVC: premature ventricular contraction; VT: ventricular tachycardia; VF: ventricular fibrillation; IL-1β: interleukin-1β; TNF-α: tumor necrosis factor-alpha; DHE: dihydroethidium. *: $p<0.05$. **: $p<0.01$
