# Supplementary material for: Binge Drinking During Adolescence Induces Long-Lasting Oxidative Stress and Inflammation in the Heart and Promotes Cardiac Dysfunction in Rats
Source: Antioxidants (Basel). 2026 Oct 21;15(10):1360. doi: 10.3390/antiox15101360 (PMC13701238; doi:10.3390/antiox15101360)
Supplement: Supplementary file 1 [file antioxidants-15-01360-s001.zip › Supplementary Table S4.pdf]

Supplementary Table S4. Mean $2^{-\Delta\Delta Ct}$ values of RT-qPCR quantifications.

Target	Control (n = 6)	ABD (n = 6)	p-value
TNF-α	0.96 ± 0.05	1.77 ± 0.30	*
IL-1β	0.92 ± 0.17	2.92 ± 0.55	**
IL-6	0.81 ± 0.19	3.17 ± 0.74	*
SOD2	1.00 ± 0.33	0.54 ± 0.14	0.1808
NOX2	1.00 ± 0.44	2.15 ± 1.88	0.3537
TGF-β1	0.79 ± 0.22	1.51 ± 0.16	*
IL-10	1.05 ± 0.14	0.74 ± 0.09	0.052

Values presented as mean ± SEM. p-values derived via unpaired t-tests with Welch's correction. *$p<0.05$, **: $p<0.01$

-
